# Phenolic Compounds: From Traditional Uses to Innovative Applications and Everything in Between

**DOI:** 10.1002/mnfr.70478

**Published:** 2026-04-28

**Authors:** Marcela de Sá Barreto da Cunha, Cristina Maria Turim Santos Miguel, Emília Maria França Lima, Uelinton Manoel Pinto, Cláudia Vieira Prudêncio, Svetoslav Dimitrov Todorov

**Affiliations:** ^1^ Center For Biological and Health Sciences Federal University of Western Bahia Barreiras Bahia Brazil; ^2^ Laboratório de Microbiologia de Alimentos Food Research Center (FoRC) Departamento de Alimentos e Nutrição Experimental Faculdade de Ciências Farmacêuticas Universidade de São Paulo São Paulo Brazil

**Keywords:** bioactive compounds, bioactivity, antimicrobial mechanisms, functional foods, sustainable applications

## Abstract

Phenolic compounds are a versatile class of bioactive molecules with growing applications in different areas, including the food and health sectors. This comprehensive review deals with many aspects related to the study of phenolic compounds, starting with advances in extraction, detection, and quantification methods, going through bioavailability, bioactivity, and beneficial health properties, and discussing antioxidant and antimicrobial uses and mechanisms. The work also discusses strategies related to sustainable use and production with opportunities related to the bioeconomy. Industrial applications include food conservation, active packaging systems, functional foods, nutraceuticals, cosmetics, and pharmaceuticals. Relevant mechanisms of action include destabilization of cellular membranes, enzymatic inhibition, oxidative stress induction, and interference on quorum sensing communication systems, with the potential to be used in anti‐virulence approaches. The potential use of phenolic compounds against planktonic and sessile bacterial cells (biofilms) is discussed, as well as their synergistic interactions with antibiotics and bacteriocins, aligned with hurdle technology in the food industry. Phenolic compounds are promising sustainable and innovative alternatives in the food, health, and industrial realms.

## Graphical abstract

This review deals with many aspects related to the study of phenolic compounds, starting with advances in extraction, detection, and quantification methods, going through bioavailability, bioactivity, and beneficial health properties, and discussing antioxidant and antimicrobial uses and mechanisms. Pointing them as promising alternatives in the food, health, and industrial realms.

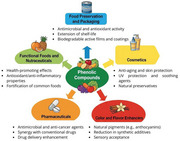

## 1 Preamble

Phenolic compounds, a miscellaneous group of secondary metabolites, are found in abundance in plants where they play important ecological roles. However, they have earned significant scientific attention and promising applications for their multifaceted roles in both the food industry and healthcare. These compounds, which include flavonoids, phenolic acids, tannins, and lignans, are honored by academia and industry for their potent antioxidants, antimicrobial, and anti‐inflammatory properties, suggesting them as invaluable in promoting human health and enhancing food quality.

One of the main applications of phenolic compounds is associated with their antioxidant activity. They can neutralize free radicals, which are unstable molecules that can damage cells and contribute to chronic diseases such as cancer, cardiovascular disorders, diabetes, and neurodegenerative conditions, including Alzheimer's and Parkinson's [1, 2]. Moreover, epidemiological studies have consistently linked diets rich in polyphenols, such as those found in fruits, vegetables, tea, coffee, wine, and whole grains, with reduced risk of chronic illnesses. It has been suggested that flavonoids like quercetin and catechins have positive effects on lowering blood pressure and improving vascular function. For instance, phenolic acids such as caffeic and ferulic acid exhibit promising anti‐carcinogenic properties, associated with modulation of enzyme activity and gene expression involved in tumor development [1].

Gut health is an additional area where phenolic compounds have shown their beneficial properties, as they can influence the composition and activity/performance of gut microbiota, enhance digestion, nutrient absorption and contribute to immune regulation and metabolic health. In the food industry, phenolic compounds play essential role as natural preservatives, flavor enhancers, and functional ingredients. Their antimicrobial properties have been explored as inhibitors of spoilage organisms and foodborne pathogens, effectively contributing to extending shelf life and ensuring food safety [3]. This is particularly appreciated for creating minimally processed or organic foods, where synthetic additives are avoided according to the demands of consumers and trends for healthier foods.

Phenolic compounds are essential for the sensory qualities of food, including color, taste, and aroma. Anthocyanins provide vibrant hues to berries and grapes, while tannins add astringency to wine and tea. These attributes not only enhance consumer appeal but also signal the presence of health‐promoting compounds.

In the last decade, the application of phenolic compounds in active packaging has been extensively explored. By incorporating phenolics into packaging materials, manufacturers can create systems that release antioxidants or antimicrobials over time, protecting food from oxidation and microbial contamination. This approach aligns with the growing demand for sustainable and health‐conscious packaging solutions [3].

Sustainability and health‐promoting lifestyle are a crossing point where phenolic compounds and the food industry met in the last decade. Extracted from agricultural byproducts like grape skins, olive pomace, and tea leaves, phenolics offer a sustainable way to valorize waste and reduce environmental impact. This not only supports circular economy principles but also aligns with ethical and ecological values embraced by modern consumers. However, despite their benefits, the application of phenolic compounds faces challenges related to stability, bioavailability, extraction efficiency, and academic and industrial challenges. Phenolics can be degraded during processing or storage, and their absorption in the human body may be limited. To overcome these hurdles, researchers are exploring encapsulation techniques, novel extraction methods, and synergistic formulations that enhance efficacy and shelf stability [2].

As trends in science and potential applications in the industry, the integration of phenolic compounds into personalized nutrition, functional foods, and preventive medicine holds immense promises and opens new avenues for applications and innovations. As scientific understanding deepens, these natural molecules may become central to strategies aimed at improving public health and food quality.

## 2 Extraction and Analysis Methods: From Tradition to Innovative Analytical and Industrial Inventions

Extracting phytochemicals from plant samples has been and is a crucial step for obtaining and characterizing bioactive compounds with potential for application in pharmaceutical and biopreservation industries. As one of the first steps, the appropriate selection of extraction methods is essential for the subsequent identification of bioactive compounds, as it can enable the obtaining of extracts with adequate concentrations of the target compounds, facilitate their transformation into more accessible and suitable forms for detection and separation, and ensure reproducibility and consistency of results, even in the presence of variations in the sample matrix [4, 5, 6].

**FIGURE 1:**
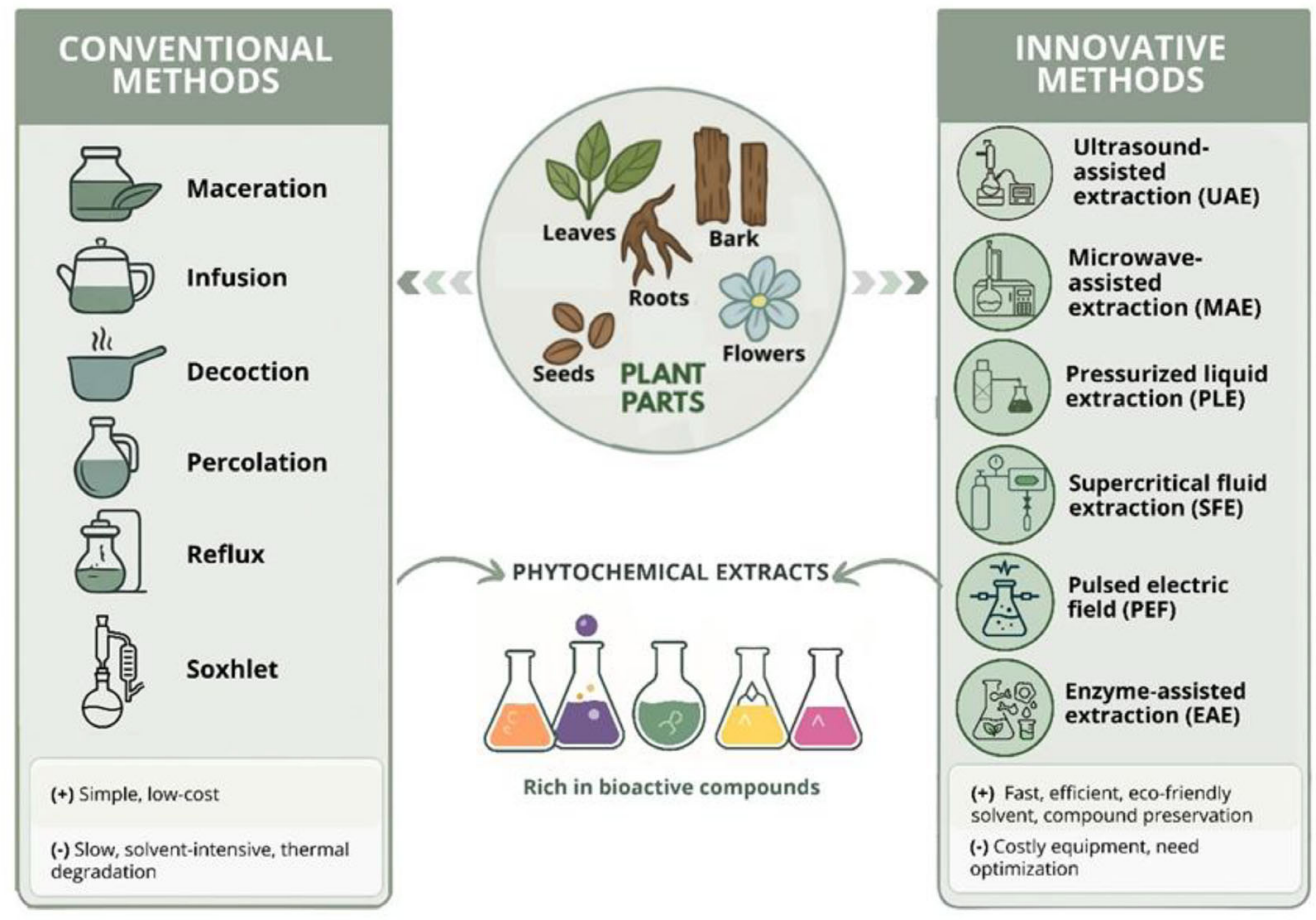
Comparison of conventional and innovative extraction methods from plant parts to obtain phytochemical extracts. Conventional methods are simple and low‐cost but slow and solvent‐intensive, while innovative methods are faster, efficient, and eco‐friendly, though requiring costly equipment and optimization. Illustration created using AI‐assisted design tools.

Conventional phenolic extraction methods have been employed for years to separate bioactive substances from diverse plant materials. These techniques can be divided according to their principles, such as immersion‐based methods—which include maceration, infusion, decoction—or continuous solvent flow/solvent recycling through reflux—percolation, Soxhlet, and reflux extraction (Figure 1) [7, 8]. Infusion and decoction are techniques that utilize hot immersion to extract hydrosoluble compounds. However, these techniques have only limited applications, related to the potential degradation of bioactive compounds during heating processes, specifically those concerning anthocyanins and catechins [8, 9, 10]. Moreover, maceration can be considered as a simple and low‐cost conventional extraction method performed at ambient or moderate temperature, but it requires long‐term extraction [4, 8]. Despite the technological obstacles, these conventional techniques remain frequently used in preliminary screening of bioactive compounds from plant material [11].

Percolation is a widely applied technique for tincture preparation, wherein the solvent gradually and continuously permeates the herb and facilitates a prolonged extraction process. This method is particularly appropriate for large‐scale obtention of herbal extracts [8, 11]. Reflux offers superior mass transport and a reduced volume compared to percolation. This approach allows for simultaneous processing of multiple samples, making it appropriate for industrial‐scale production, but it is inefficient for non‐volatile chemicals and is mostly employed to identify flavonoids and saponins [7, 8]. A recent study on hemp (*Cannabis sativa*) seed examined the optimization of reflux extraction parameters, including time, liquid‐to‐solid ratio, particle size, and solvent concentration, resulting in enhanced yields of total phenolic and flavonoid content, substantial quantities of flavonoids, and a reduction in inflammatory markers [12].

Soxhlet extraction utilizes heated solvents that continuously circulate through the plant material, recovering the target compounds in a concentrated extract. Despite its extensive applicability, the disadvantages are associated with the prolonged extraction times and the possible degradation of compounds at high temperatures [7, 11, 13]. In a study with tea plant (*Camellia sinensis*) leaves, Soxhlet extraction resulted in the highest yield compared to other conventional methods, reaching 40.6% of the initial plant mass of the extract, especially total flavonoids [14].

The specific part of the plant used is also important when choosing the best extraction method. For instance, infusion is generally better for soft parts like flowers, leaves, and tops, while decoction is usually employed to extract compounds from tougher parts such as roots, bark, seeds, hard stems, and rhizomes (see Sentkowska et al. [15] for more details). Moreover, the challenges in the preparation of plant extracts is to promote higher yield without raising costs, to reduce time in extraction, to respect the sustainability, to use of eco‐friendly solvents and reduce energy costs. All of these require the development of innovative extraction techniques. Novel extraction methods involve combination of traditional techniques with innovative ideas like ultrasound‐assisted extraction (UAE), microwave‐assisted extraction (MAE), pressurized liquid extraction (PLE), supercritical fluid extraction (SFE), pulsed‐electric field treatment (PEF), and enzyme‐assisted extraction (EAE) (Figure 1) [10, 16].

In UAE approach, the plant cell walls are disrupted, increasing cell permeability and the contact area between solvent and sample. As this extraction occurs at lower temperatures compared to Soxhlet, for example, thermolabile compounds such as carotenoids, vitamin C, and some phenolics are preserved. This approach reduces extraction time, energy consumption, and solvent usage compared to conventional methods, such as maceration, which configures as an important technological advantage. However, this process generates many reactive oxygen species concomitants with the creation of cavitation bubbles, potentially leading to the oxidation or deterioration of some compounds [11]. The MAE approach can facilitate direct interactions between microwaves and polar compounds, significantly decreasing extraction time and enhancing recovery efficiency for thermostable compounds; nonetheless, its main limitation is for the extraction of lipophilic compounds unless cosolvents or matrix pre‐wetting are used [17].

Some of the previously mentioned innovative extraction techniques were applied by El Maaiden et al. [18], who reported on the influence of extraction methods on the pharmacological and cosmetic properties of six medicinal and aromatic plants: *Corrigiola litoralis* (L.), *Tamarix aphylla* (L. H.), *Pistacia lentiscus* (L.), *Cyperus rotundus* (L.), *Nardostachys jatamansi* (D. Don, DC.), and *Lavandula coronopifolia* Poir. Some of the advanced techniques were identified to improve yield for all evaluated plants relative to conventional methods, underlining that MAE approach can lead to greater yield of bioactive compounds. Furthermore, when focus was on obtaining total phenolic and total flavonoid contents, UAE technique emerged as the more advantageous method. Although the overall yield was relatively low, conventional methods showed higher levels of total anthocyanin recovery and antioxidant activity. Among these methods, infusion was the most effective both for anthocyanin quantification and for antioxidant activity, followed by decoction, maceration, and UAE. Moreover, Abutayeh et al. [19] recently reported on antimicrobial potential of pomegranate peel extracts with effective inhibitory properties against *Staphylococcus aureus*, *Escherichia coli*, and *Pseudomonas aeruginosa*, obtained by maceration, infusion, decoction, and MAE.

The PLE technique has advantages that can reduce the use of solvents, operate with high temperatures and pressures, and is a rapid and efficient extraction process. The extraction is optimized when the solvent polarity is chosen based on the polarity characteristic of the target compound, facilitating the solubilization and minimizing the co‐extraction of interferences [20]. Hollas et al. [21] used PLE with a nonpolar solvent to recover oil from rice bran, achieving high extraction yields and efficient conversion to biodiesel.

Another extraction method based on fluid under pressure is SFE, that uses fluid above its critical temperature and pressure, commonly supercritical CO_2_. It is a simple extraction process, related to the low solvent consumption, and high extraction yields, which enhances the feasibility of using this method at an industrial scale. Despite these advantages, additional steps may be required for some compounds, once supercritical CO_2_ mainly extract low‐polarity compounds, so it is necessary to use co‐solvents, such as ethanol, methanol, and acetone [7]. Moreover, combined extraction with SFE and PLE allows the recovery of both hydrophilic and lipophilic compounds, enriching the fraction with different bioactive compounds, such as tocopherols, fatty acids, and phenolic compounds [22, 23].

PEF is a nonthermal extraction method that uses very short electric fields and has applications in food processing, tissue disruption, and bioactive compound extraction. It works by destabilizing the cell membrane, increasing its permeability, and facilitating intracellular extraction. The advantages are related to the absence of aggressive chemical eluents/products and the use of water as the solvent, considered a green extraction method that contributes to sustainable food [24, 25].

The application of PFE as an extraction method was suggested to investigate the relationship between electric field intensity and pulse frequency on the properties of avocado (*Persea americana*) seed extract, focusing on its antioxidant and antibacterial activities, as well as its phenolic (benzoic acid and dihydrophenol) and fatty acid composition. High antioxidant capacity (95.56% DPPH inhibition, IC_50_ = 90 µg/mL) and antibacterial effects against *E. coli* and *S. aureus* were reported [26].

The principles of EAE method are based on the enzymatic hydrolysis of plant cellular membranes, enhancing the permeability and compound release [27] and this can be applied in improvising yield of plant‐based extracts. It has been suggested that this extraction approach can be applied as a stand‐alone technique or as a pretreatment for traditional extractions [28, 29]. Moreover, EAE can be considered a highly specific method, since enzymes are selected to degrade the plant matrix, preserving many classes of bioactive compounds, such as phenolics, carotenoids, terpenes, proteins, and fatty acids [27]. Furthermore, the advantages are related to its relatively low cost and sustainability, through a reduction in the use of toxic solvents and the recovery of compounds from agricultural residues [27, 28].

Recovery and yields of phenolic compounds extracted from plant matrices depend not only on the applied technique, but also on the chemical nature and binding forms of these compounds. Taking this into account, the right selection of solvents, extraction conditions, and pretreatment steps are critical for optimizing phenolic compounds recovery, while at the same time minimizing compounds degradation [15, 30]. A study performed with the leafy stems and roots of *Phyllanthus amarus* compared maceration extraction using acetone‐water and ethanol‐water mixture, and it was found that acetone‐water provided a better total polyphenol extraction from leafy stems, while ethanol‐water mixtures were more effective in recovering flavonoids from roots [31]. A study conducted by Lima et al. [32] investigated the differences in the influence of various extraction methods (maceration, sonication, infusion, decoction, and microwave extraction) on the phytochemical profile and biological potential of extracts from *Ageratum conyzoides*, *Plantago major*, and *Arctium lappa* L. Regarding antioxidant activity, although infusion demonstrated superior complexation capacity, no significant differences were observed among the decoction, infusion, and microwave methods. However, sonication was the most effective method, yielding extracts with higher antioxidant activity (> 90% for the *Arctium lappa* extract) and a high phenolic content, evidencing a strong correlation between these parameters. Additionally, sonication‐derived extracts showed a high flavonoid content (298 mg for *A. conyzoides*, 154 mg for *A. lappa* L., and 122 mg for *P. major*). Thus, these are just a few examples that clearly highlight the relevance of choosing methods based on the specific phytochemical class and the intended biological activity.

## 3 Detection and Quantifications Methods of Phenolic Compounds

**FIGURE 2:**
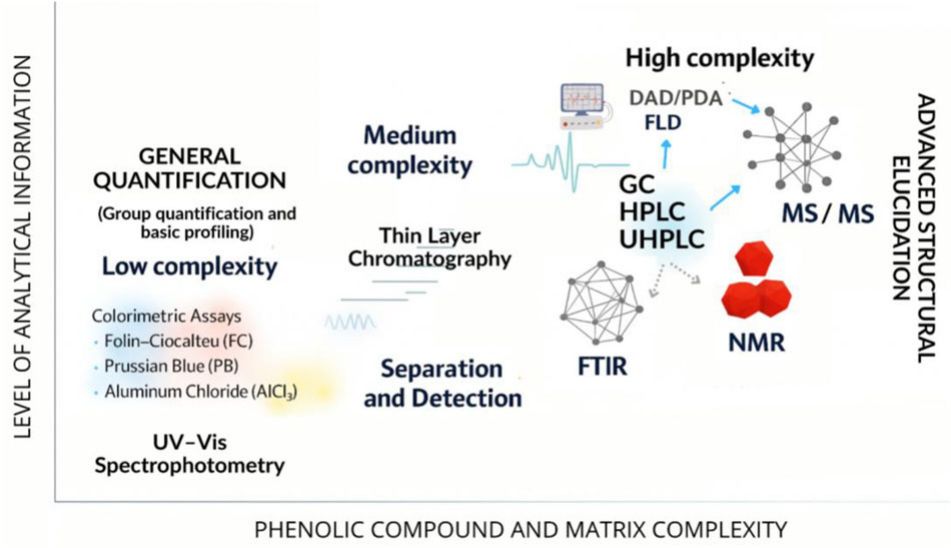
Comparative overview of analytical techniques for phenolic compound analysis. The figure relates analytical depth (y‐axis) to compound and matrix complexity (x‐axis). Simpler assays, such as colorimetric and UV–vis, provide general quantification; intermediate techniques like TLC offer separation and profiling. Chromatographic approaches (GC, HPLC, UHPLC) combined with detectors (e.g., DAD, FLD) or mass spectrometry (MS/MS) enable targeted and structural analysis. FTIR and NMR allow advanced structural elucidation, especially post‐separation. Illustration created using AI‐assisted design tools.

Various analytical technologies are available for the detection and structural elucidation of phenolic compounds. These include UV–visible (UV–vis) spectroscopy, fluorescence (FL) spectroscopy, infrared (IR) spectroscopy, nuclear magnetic resonance (NMR) spectroscopy, and mass spectrometry (MS). Among these, MS is widely used as a primary tool for the characterization of phenolic compounds. These methods can be utilized independently or coupled with chromatography techniques, which allow the separation of compounds and consequently provide more specific compositional information [33, 34, 35, 36, 37]. These techniques vary widely in their sensitivity and specificity. Figure 2 illustrates the relationship between phenolic compound complexity, matrix characteristics, and the level of analytical information obtained using different methods.

The Folin–Ciocalteu (FC) assay is a colorimetric method widely used to quantify total phenolic content (TPC) in plant extracts. The FC reagent turns blue when it reacts with phenolic compounds, and the intensity of this blue color (measured between 725 and 750 nm) indicates the amount of phenolic content in the extract [33]. Another color test is the Prussian Blue (PB) assay, which detects phenolic compounds by using a blue reagent. A blue complex forms when antioxidants in the sample reduce iron. Similar to FC, a more intense color indicates a higher concentration of phenolic compounds present. Although both procedures are fast and simple, they lack specificity to differentiate classes of phenolic compounds and present low sensitivity. Their accuracy may vary depending on the plant matrix and the solvent chosen; its accuracy may vary [38].

Considering phenolic subclasses, flavonoids can also be detected using a colorimetric test based on aluminum chloride, in which aluminum ions form complexes with flavonoid hydroxyl groups. The color of this complex is measured by spectrophotometry at 510 nm. While FC and PB react widely with phenolic compounds, aluminum chloride complexation occurs preferentially with hydroxyl groups in the catechol B‐ring. Thus, it is possible to determine flavonoids in a plant matrix, minimizing interference with other phenolic compounds [38].

To obtain precise information on individual phytochemicals, especially in complex food matrices, chromatographic methods are frequently applied. These techniques enable the detection of phenolic and other bioactive compounds, separation, and quantification [37, 39]. High‐performance liquid chromatography (HPLC) with UV detection (HPLC‐UV) is one of the most used analytical methods due to its reliability, well‐established methods, and wide range spectral library. The principle of chromatography is based on the separation of compounds due to their different affinity for two phases: a stationary phase and a mobile phase [39]. Moreover, gas chromatography (GC) is widely used to detect volatile and thermo‐stable compounds. Its principle relies on the separation of analytes based on their volatility and interactions with the stationary phase [39].

Thin‐layer chromatography (TLC) is a simple method that separates compounds based on their interaction with a stationary phase, a thin adsorbent layer on a plate, and a mobile phase, a solvent that migrates upward by capillarity. Separation occurs based on the differential affinity of the compounds for the stationary and mobile phase. TLC is mostly used for initial screening and qualitative detection of phenolic compounds, as it presents lower sensitivity and sensibility compared to HPLC and GC [37, 39].

To obtain more accurate information on phenolic compounds, especially in complex matrices, more advanced chromatographic methods were developed, as well as hyphenated techniques. Ultra‐high‐performance liquid chromatography (UHPLC) is an evolution of traditional HPLC that utilizes smaller particles and higher pressures, which culminates in a faster separation with higher sensitivity. In the food context, UHPLC is mostly used in the detection of contaminants and quantitative analysis of bioactive compounds in complex matrices [40, 41]. Hydrophilic interaction liquid chromatography (HILIC) is a type of liquid chromatography that is used to separate highly polar compounds that are usually retained in conventional chromatography, such as water‐soluble vitamins and polyphenols in dietary supplements and plant‐derived food [42, 43].

In chromatographic analytical and preparative analysis, the selection of an appropriate detector is essential to guarantee the identification and quantification of phenolic compounds precisely. The detection methods vary based on sensitivity, compatibility with different analytes, and the mobile phases used [39, 44]. Detectors UV–vis, including diode array detector (DAD) and photodiode detector array (PDA), are the main ones utilized in phenolic analysis. These detectors monitor the absorbance in a wavelength range, allowing multiple compounds to be detected with distinct spectral properties. Most of the phenolic compounds’ absorbers are in the UV region (between 200 and 400 nm), in which detection is efficacious even in low concentrations. DAD and PDA are techniques that provide valuable spectral fingerprints that help in identification when compared to standard spectral data [44, 45].

Fluorescence detectors provide more sensibility than UV–vis for some classes of phenolic compounds, such as flavonoids that present an intrinsic fluorescence [46]. This method allows detection at trace levels, and it is useful in samples with low phenolic content or with high background [47]. Ferreyra et al. [48] used fluorescence to quantify flavanols, stilbenes, and phenyl ethanol analogues, achieving enhanced sensitivity compared to conventional DAD detection. Moreover, the authors suggested that their method precisely quantified flavanols, stilbenes, and phenyl ethanol analogues in different winemaking products.

Mass spectroscopy, when coupled to chromatographic approaches such as liquid chromatography (LC‐MS), GC‐MS, or UHPLC‐MS/MS, provides high sensitivity structural elucidation and quantification [49]. It operates based on the ionized analyte ratio mass/charge (*m/z*). These characteristics permit enhanced selectivity through identification of specific mass fragments, high sensitivity capable of detecting phenolic compounds in nano or even picograms, and structural analysis, especially in mass in tandem (MS/MS) [39].

When applying LC‐DAD‐MS, de Araújo et al. [50] were able to identify 27 compounds in the pulp of *Campomanesia adamantium*, a native fruit from Brazilian Cerrado, including phenolic compounds, flavonoids, and organic acids. In the context of in vivo analysis, the fruit extract prevented oxidative stress and promoted increased longevity in a *Caenorhabditis elegans* model, and observations from the authors allowed a link of biological functions with detected bioactive compounds.

Coutinho et al. [51] applied HPLC‐MS and identified and quantified anthocyanins, such as cyanidin‐3‐glucoside and cyanidin‐3‐rutinoside, in white açaí (*Euterpe oleracea* Mart) extract, indicating that the composition and concentration of these bioactive metabolites are in lower amounts when compared to purple açaí.

In the context of applied GC‐MS, Pascoal et al. [52] analyzed in detail the profile of volatile and metabolite compounds in different varieties of Brazilian *pitanga* (*Eugenia uniflora* L.) in various stages of fruit ripening. The results showed different bioactive compounds presented in *pitanga*, including esters, terpenes, aldehydes, fatty acids, and sugars, indicating chemical variation during ripening that influences the sensory properties and beneficial properties of the fruit [52].

Moreover, other analytical methods can be used to complement the characterization of phenolic compounds in plant matrices. Fourier‐transform infrared spectroscopy (FTIR) can contribute to the knowledge of how a studied compound can absorb infrared radiation at different wavelengths, allowing the confirmation of phenolic compounds based on the characteristic absorption bands of the functional group. Nevertheless, it presents low sensibility and a low applicability in complex mixtures [53].

Nuclear magnetic resonance (NMR) spectroscopy provides detailed structural information and is widely used in the elucidation of complex phenolics [54]. One of the main advantages is that it requires minimal sample preparation, allowing the use of liquids, solids, and gases, making it a valuable tool in the characterization of complex matrices and natural products [55]. Wu et al. [56] demonstrated NMR application in the identification of fifteen compounds including phenolic acids and terpenes, in *Salvia miltiorrhiza*. For some foods such as wheat, rice and corn, its combination with GC‐MS and LC‐MS have been an essential tool in diverse research areas to identify and quantify a broad range of bioactive compounds [57].

## 4 Bioavailability and Bioactivity

The role and benefits of phenolic compounds in humans and other animals have been extensively investigated over the past few decades [58, 59, 60], and evidence extensively demonstrates that their effects are primarily determined by two key factors, bioavailability and bioaccessibility.

Bioavailability refers to the extent and rate at which a compound reaches systemic circulation and becomes available at the site of action. It is a complex process where phenolic compounds are assessed, involving their digestion, solubilization, absorption through the gastrointestinal tract (GIT), and further metabolic transformation [61]. Moreover, with the objective of being adsorbed by epithelial cells, those compounds must be initially released from the food matrix during digestion processes, a process defined as bioaccessibility, which corresponds to the readily accessible fraction of the compound available to absorption [62].

Different factors can influence phenolic compound bioavailability, including matrix interactions, host physiology, phenolic structural characteristics, and numerous external factors [61, 63]. The presence of hydrophobic groups in some phenolic compounds can make the solvent penetration more difficult, as can be associated with reduced solubility, and therefore, it can compromise the bioavailability [64]. Moreover, interaction with different food components, including fibers and macromolecules, can also affect their absorption and availability. Diez‐Sánchez et al. [65] reported that polyphenols from blackcurrant (*Ribes nigrum*), when bound to dietary fiber, can reduce their release and bio‐accessibility. Among macronutrients, protein was frequently pointed out as the food component with the most protective effect, as it can be involved in the interaction processes that will protect phenolic compounds from degradation during digestion, and increase phenolic compounds bioaccessibility.

Systemic host factors, such as age, gender polymorphism, nutritional status, gut microbiota, are just some of the factors that can influence the phenolic compounds metabolization and can modulate their biological role [66, 67]. In a study with prepubescent rats, it was observed that gender differences between the male and female can play a role in the recorded concentration of procyanidin of grape seed and some metabolites in liver white adipose tissue and kidneys [68]. Fraga et al. [69] explored the polymorphisms in genes associated with the flavones transport and metabolization (SULT1A1, SULT1C4, and ABCC2) and reported on interindividual variability in processes of absorption and metabolization after orange juice ingestion. Moreover, Mena et al. [70] stated that healthy volunteers who ingested green tea extract and green coffee exhibited different urinary excretion profiles, influenced by microbial production of flavan‐3‐ols derivative.

One of the topics explored in the last decades was associated with improving the bio‐availability of the beneficial metabolites, including polyphenols. Traditional strategies in this direction involve food processing aiming to reduce antinutritional components. However, such processing methods present some limitations, such as degradation or structural alteration of phenolic compounds [71]. Treatments involving high temperatures, the process of lyophilization, or even different culinary methods are the main factors that can alter bioavailability; e.g., curcumin is unstable under prolonged heat and alkaline pH, being degraded during the cooking process, which may reduce its concentration to absorption [72]. It is important to note that curcumin was used as a representative case study due to the depth of available literature and its relevance to multiple sections of the current review.

In the last decades, several innovative approaches were suggested and applied, including nanotechnologies. Nano‐delivery systems are techniques that can release structured bioactive compounds on a nanometric scale, with the objective to protect, transport, and control the release of these compounds, enhancing their stability, solubility, bioavailability, and biological activity [71]. Nano‐carriers can be composed of specific lipids, polysaccharides, proteins, or their complexes and include nanoparticules (NP), nanohydrogels (NH), nanoemulsions (NE), nanoliposomes (NLP), exosomes, cyclodextrin, and micelles [64].

Nanoparticles may be derived from starch granules (e.g., from potato, corn, or rice) or synthesized from simple substances like zein [64]. As examples, nanohydrogels can be useful for the delivery of both hydrophobic and hydrophilic compounds, like chitosan‐based NH, which show high capacity of swelling in acidic environment, being suitable for gastric delivery and may even act as prebiotics [73].

Since polyphenols generally present low lipid solubility, the formulation of NE is one of the strategies to increase dispersion and absorption. Moreover, NE are thermodynamically stable, homogeneous systems formed spontaneously by water, oil, surfactants, and/or co‐surfactants. The most common types of NE are water in oil (W/O), oil in water (O/W), and water in oil in water (W/O/W). The antioxidant properties of these emulsions are influenced by the type and the stability of the emulsion and the phenolic concentration [74].

NLP are composed of lipid bilayers and are utilized to co‐encapsulate multiple phenolic compounds, such as curcumin (hydrophobic) and oligomeric proanthocyanidins (more hydrophilic). These structures protect the compounds from degradation and improve their physical stability and bio‐accessibility, thereby enhancing cellular absorption. The choice of phospholipid, such as soybean phospholipid, is essential, as it influences particle size, homogeneity, and the antioxidant capacity of NLP [75].

**FIGURE 3:**
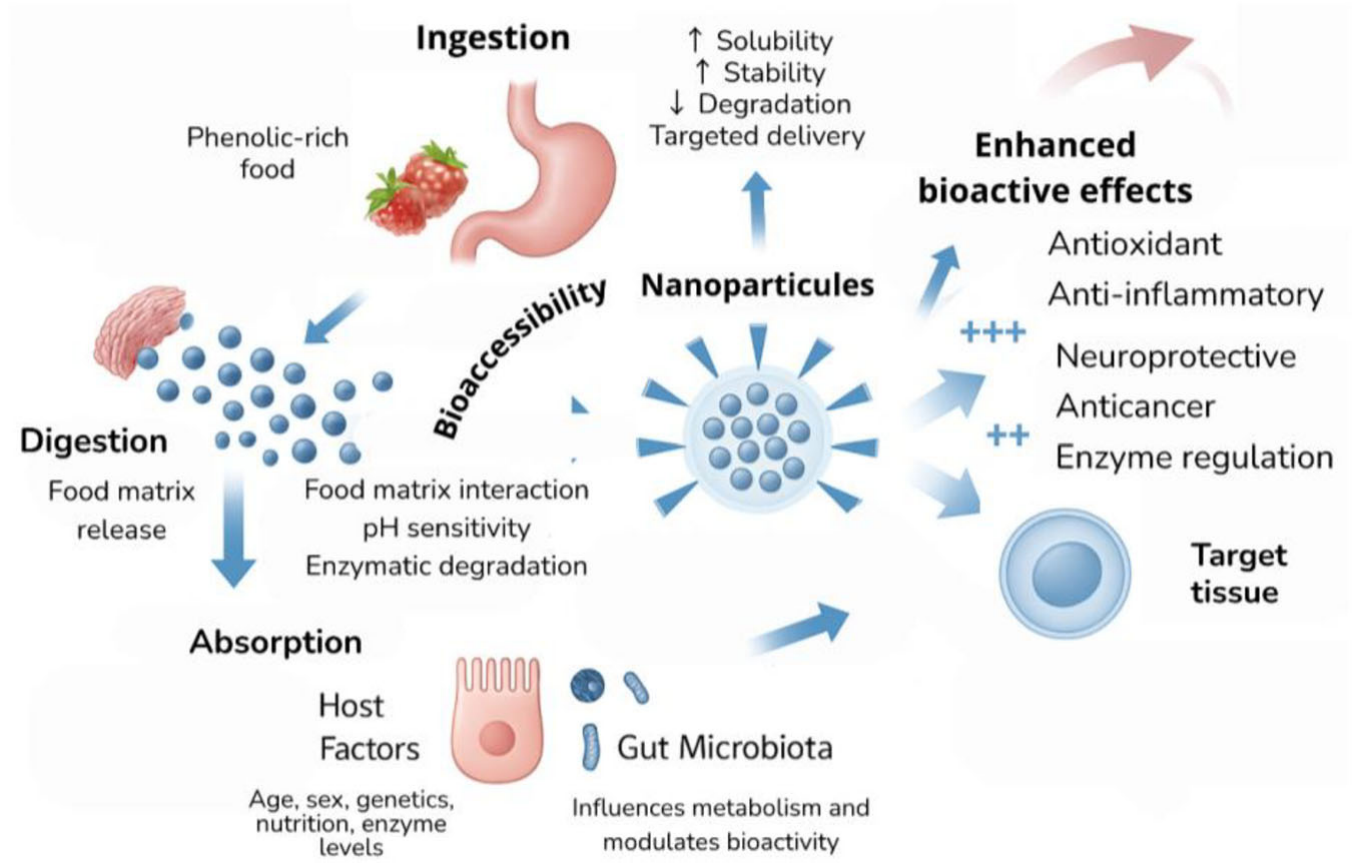
Overview of phenolic compounds’ absorption and enhanced bioactivity via nanotechnology. Nanoformulations improve solubility, stability, absorption, and targeted delivery, enhancing bioavailability and physiological effects. Illustration created using AI‐assisted design tools.

Micelles and cyclodextrins are highly efficient in improving the solubility and chemical stability of hydrophobic phenolics. Micelles are formed by the self‐assembly of hydrophilic molecules, whereas cyclodextrins encapsulate bioactives within their hydrophobic cavity. Additionally, exosomes generated from plants are a novel approach for the natural, targeted administration of polyphenols, providing good biocompatibility and protecting from degradation during gastrointestinal transit [64, 71]. Nanotechnology is not only linked to the increase of the phenolic compounds’ bioavailability, but also its influence on their bioactivity. When an active metabolite arrives at target‐tissues, antioxidants, anti‐inflammatory or neuroprotective effects are also potentialized, as shown in Figure 3.

Abdel‐Mageed et al. [76] suggested incorporation of curcumin into cyclodextrin and generation of hybrid nanoparticles (NP) for potential applications in wound healing. Generated NP presented robust anti‐inflammatory and broad‐spectrum antibacterial activity, inhibiting all tested strains (*S. aureus, Bacillus subtilis, E. coli, and Pseudomonas aeruginosa*), with particularly higher efficacy against Gram‐positive bacteria.

Furthermore, in vitro assays demonstrated increased fibroblast growth and movement, while in vivo studies in a burn rat model showed faster wound closure and tissue regeneration. Moreover, Long et al. [77] investigated the role of the rosmarinic acid in a DSS‐induced colitis mouse model and reported that this phenolic compound, when applied in nano‐formulation, can present high radical scavenging and attenuated colonic inflammation, suggesting potential therapeutic application in this pathology. An additional example can be the application of quinoa protein‐based NP with effects for improving the bioactivity of buriti oil and even enhancing its antioxidant potential and enzymatic inhibition capacity. It was also demonstrated that nano‐formulations may play a positive role in increasing the solubility, stability, and delivery of lipophilic compounds, with significant inhibition of α‐amylase and α‐glucosidase, a beneficial characteristic for the potential antidiabetic property [78].

Dietary phenolic compounds bioactivity is directly linked to their bioavailability, which depends on intestinal absorption, subsequent metabolic transformations, and distribution to target tissues [79]. Since these compounds present low bioavailability, their bioactivity is often mediated by metabolites, especially those produced in gut microbiota and particularly to the hepatic metabolism [79, 80]. For example, the post‐ingestion metabolism of wheat bran‐bound ferulic acid showed low bio‐accessibility (1.7%) and systemic absorption (0.3%); however, fecal metabolomic analysis showed colonic bioconversion into ferulic‐acid bioactive metabolites [81]. A transversal study with older adults showed that urinary levels of microbial phenolic metabolites were closely associated with a better cognitive fucntion, corroborating the role of gut microbiota in neuroprotective bioactivity of polyphenols, by bioconversion [82].

## 5 Health Benefits and Applications

Phenolic compounds have long been used in traditional medicine, and modern science has identified their benefits and thoroughly studied their mechanisms of action over the past century. Scientists generally agree that phenolic compounds serve as antioxidants, helping to neutralize free radicals and reduce oxidative stress, which in turn protect the integrity of biomolecules. Taking into consideration that those processes are closely related to cellular aging and chronic disease, such as cardiovascular disease, cancer, and cognitive function, pointing clearly to health‐promoting benefits for phenolic compounds [58, 59, 83].

It was shown that some classes of phenolic compounds can also present anti‐inflammatory properties, attenuating systemic and local inflammatory processes. An authority review showed that *Moringa oleifera* Lam. (Moringaceae), which is known as a great source of flavonoids, alkaloids, glucosinolates, tannins, and phenolic acids, tended to downregulate pro‐inflammatory and upregulate anti‐inflammatory pathways in vitro and in animal models studies [84]. In the context of prevention of inflammatory bowel diseases, many flavonoids present a regulatory effect on the balance of T CD4 cell subtypes, by modulating transcription factors related to immune cell differentiation [85]. There is also evidence from randomized controlled trials of diverse dietary polyphenols that provide benefits in rheumatoid arthritis by decreasing inflammation and oxidative stress, as well as improving the disease activity score [86].

Quercetin, curcumin, resveratrol, anthocyanin, cathechins, and other bioactive compounds can positively affect the cardiovascular system and even may reduce systolic blood pressure, improve blood lipid profile and glucose metabolism, and enhance cardiac function indices [87, 88, 89]. Regular consumption of foods naturally presenting or fortified with phenolic compounds, such as green tea extract‐fortified rye bread and olive fruit (poly)phenol‐fortified yogurt, also presented similar effects, associated with glucose and blood lipid levels improvement [90]. Moreover, phenolic compounds can actively influence various pathways related to cellular growth and differentiation, inducing apoptosis in tumor cells, inhibiting tumor progression, and being a promising adjuvant therapy in antineoplastic agents [91, 92, 93, 94].

Other beneficial effects can involve the improvement of gut health, including altering microbiota composition, reducing systemic inflammation, or favoring metabolic control. Microbiota modulation reinforces intestinal barriers, reducing permeability. There are also reported neuroprotective effects, acting directly in the nervous system or indirectly, by interfering with the gut–brain axis. The reduction of neuroinflammation can protect intestinal integrity and restore the host microbiome, contributing to preserving cognitive function and attenuating neurodegenerative diseases linked to oxidative stress and inflammation [95, 96, 97, 98].

It is interesting to point out that some of the emerging applications of phenolic compounds in health have been developed as initial research, mainly with focus to curcumin. Curcumin nanoencapsulation showed excellent stability and a sustainable delivery for 72 h, and offered an antiproliferative, dose and time‐dependent effect against glioblastoma (U87 MG) and colon cancer cells (Caco‐2) [99]. Moreover, another biomedical application for curcumin involves Parkinson's disease (PD), a chronic neurodegenerative condition marked by oxidative stress and damage to dopaminergic neurons. The development of a hybrid nanoparticle combining levodopa, the primary pharmacological drug for treatment of PD, and curcumin demonstrated in vitro study, that oxidative stress can be reduced and can increase neuronal survival in damaged neurons. Furthermore, in the in vivo model, administration of the newly developed nanoparticles in mice resulted in preservation of dopaminergic neurons, a reduction in oxidative stress, and no observed toxicity in the major organs assessed [100]. Besides, a recent study showed that curcumin in hybrid nanoparticles presented low cytotoxicity in human fibroblast (HFF‐1) and high antioxidant capacity in vitro. In a DSS‐induced colitis mouse model, the treatment with the NPs with curcumin reduced the disease score index, protected the intestinal mucosa against severe damage, decreased inflammatory infiltration, and preserved intestinal villi structures [101].

Considering the evidence and potential of phenolics in food and nano‐formulations, more research is needed to identify additional compounds, optimize nanoparticles, and confirm their effectiveness and long‐term safety both in vivo and in controlled clinical trials.

## 6 Sustainable Production and Use

The widespread use of phenolic compounds remains limited by the low yields and relatively high cost of extraction processes, in addition to challenges related to reproducibility. Furthermore, crude extracts may contain allergenic and/or toxic substances, which restrict their application and necessitate purification techniques—procedures that typically increase production costs even further [102]. In this context, the use of advanced “green” technologies and appropriate solvents and extraction methods that enhance yield have emerged as sustainable alternatives for increasing phenolic compound production [103, 104]. Among these, biotransformation approaches such as solid‐state fermentation (SSF) and microbial biosynthesis have shown promise.

Solid‐state fermentation involves the production of target compounds by microorganisms grown on solid substrates with low water activity. This method stands out for its high productivity and potential for using low‐cost agricultural and agro‐industrial residues as substrates, allowing for a reduction in production costs [105, 106]. Rivera et al. [107] demonstrated that SSF by *Rhizopus oryzae* increased phenolic compound content by 106.7%–176.2%, depending on the applied specific conditions. Coronado‐Contreras et al. [35] also reported enhanced tannin production following SSF by *Aspergillus* spp., as did Abduh et al. [108], who observed increased phenolic content and antioxidant activity with the use of the same microorganism. These findings highlight the potential of SSF as a viable strategy to boost phenolic compound yields, which can positively impact production costs.

Microbial biosynthesis has also emerged as a promising strategy for improving yield and scaling production, particularly through omics‐based approaches that enable the integration and modeling of genome‐scale metabolic pathways [109]. This strategy has been successfully employed to produce resveratrol using *Saccharomyces cerevisiae* [110], flavanones using *E. coli* [111], and flavones using *E. coli* [112]. However, microbial biosynthesis for phenolic compound production still has limitations, such as the need to alter metabolism due to lower flux in secondary metabolic pathways; the necessity to increase the catalytic activity of many genes given the complex molecular structure of phenolic compounds, and complex molecular structure of phenolic compounds [113].

Complementary, the circular bioeconomy proposes the use of residues that can be converted into high‐value bioproducts [114]. From this perspective, the use of residues from the agro‐food industry also promotes sustainability by proposing new use strategies for the large amount of waste generated and by reducing the demand for natural resources to produce these compounds [104, 115]. Several studies show the possibility of recovering phenolic compounds from food residues such as pomegranate, pineapple, and jaboticaba peels, grape seeds and stems, and orange and tomato peels, highlighting the broad range of raw materials that could be used [103, 107, 115, 116, 117, 118, 119].

Another important theme is the antimicrobial potential of phenolic compounds, whose use in food may contribute to the control of pathogenic and spoilage microorganisms, potentially reducing losses of large quantities of food [109]. Moreover, phenolic compounds can be extracted from native species of different biomes, such as *buriti* (*Mauritia flexuosa* L. f) and *aroeira* (*Schinus terebentifolius* Raddi), which are found in Brazilian ecosystems [120, 121]. This strategy promotes the valorization of native species and may positively impact biome conservation by fostering sustainable alternatives for their use in the production of high‐value‐added products [120].

## 7 Industrial Applications

**FIGURE 4:**
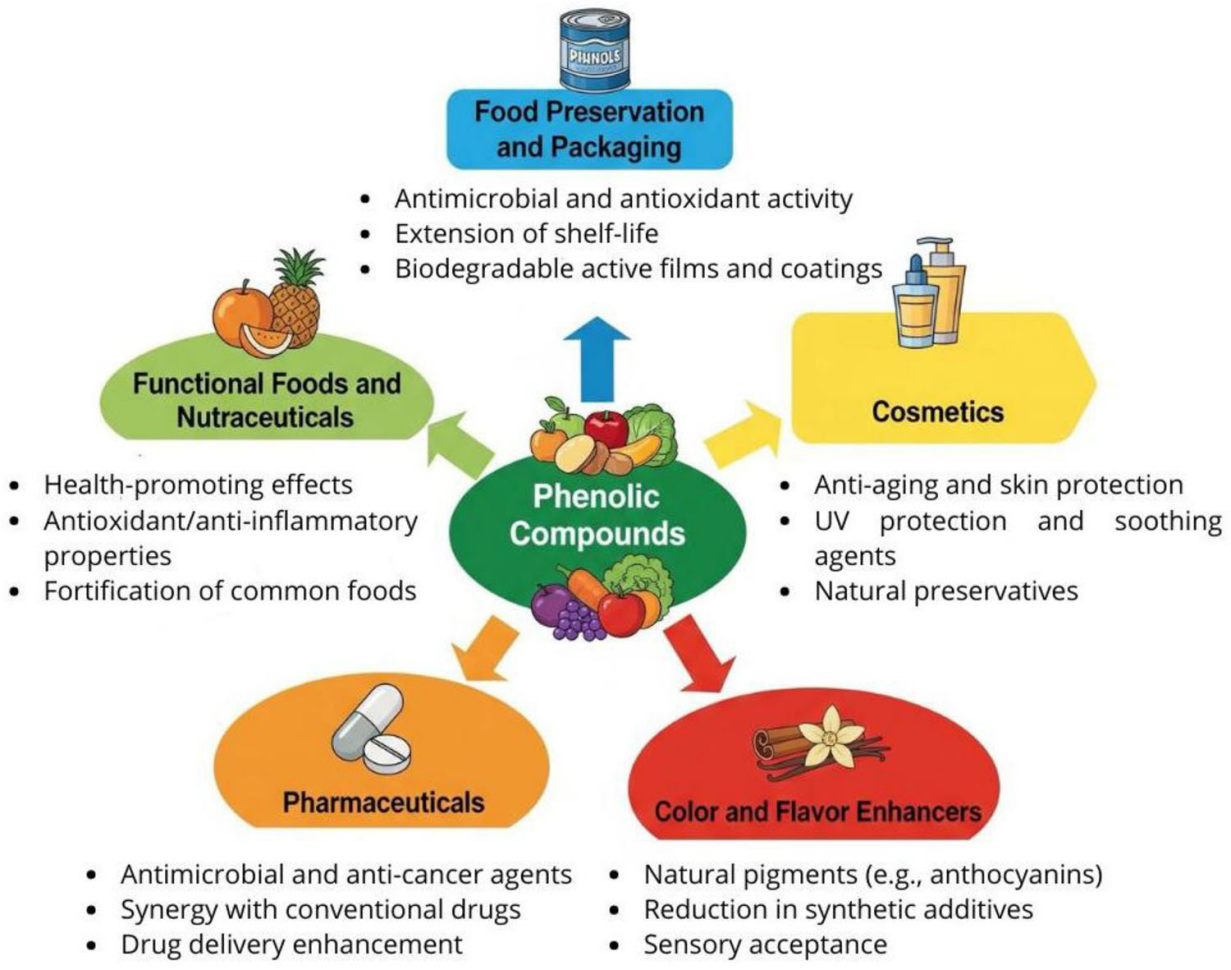
Potential industrial applications of phenolic compounds. Phenolic compounds exhibit multiple biological activities and can be used as additives or as replacements for conventional ingredients by various industries, providing functional, nutritional, and/or safety‐related improvements. Illustration created using AI‐assisted design tools.

Besides health‐promoting properties, phenolic compounds offer broad industrial applications (Figure 4), including enhancing color, flavor, and aroma of foods, along with contributions to food safety and functional benefits through their antioxidant activity [122, 123]. Beyond food applications, phenolic compounds are also employed in cosmetics and pharmaceuticals, where their antioxidant and bioactive properties support skin protection and may assist in handling various diseases [124, 125, 126].

### 7.1 Food Preservation and Food Packaging

The use of natural preservatives has a millennia‐long history, dating back to prehistoric times. However, the use of plant‐derived compounds has gained prominence since synthetic preservatives are increasingly associated with diseases and allergies [122, 127]. This shift also reflects changes in consumer behavior, with consumers becoming increasingly aware of the ingredients used in food production and generally recognizing non‐natural substances as toxic and/or harmful to health [102].

The notable antimicrobial potential of plant extracts containing phenolic compounds has been observed in materials derived from various species, including fruits such as *buriti* (*Mauritia flexuosa* L. f.), pomegranate (*Punica granatum* L.), blueberry (*Vaccinium corymbosum* L.), grape (*Vitis vinifera* L.), and *jaboticaba* (*Plinia cauliflora* L.) [117, 120, 128, 129, 130]; vegetables like radish (*Raphanus sativus* L.) [131]; and herbs, such as *Pulicaria odora*, sea fennel (*Crithmum maritimum* L.), coriander (*Coriandrum sativum* L.), and roselle (*Hibiscus sabdariffa* L.) [132, 133, 134, 135, 136].

Several studies have reported that compounds have exhibited antimicrobial activity against various bacteria, including foodborne and pathogenic species such as *E. coli, Bacillus subtilis, Bacillus cereus, P. aeruginosa, Salmonella* Enteritidis, *Listeria monocytogenes*, and *S. aureus;* yeasts such as *Candida albicans*; and fungi including *Aspergillus flavus, Aspergillus niger*, and *Trichoderma reesei* [120, 128, 129, 130, 131, 132, 133]. In this regard, the combined use of phenolic compounds with other substances can produce more significant results, as shown by Liu et al. [136], who tested gallic acid combined with gadolinium and observed enhanced bactericidal activity against multidrug‐resistant clinical isolates of *Salmonella enterica*. Moreover, the use of combination of natural antimicrobials such as phenolic compound and antimicrobial proteins (bacteriocins) to improve food safety and shelf life for food products is an active area of extensive promising research. Phenolic compounds offer antioxidant and antimicrobial benefits, while bacteriocins, peptides produced by microorganisms, specifically can inhibit bacterial growth of spoilage and pathogens without influencing beneficial species. Combining these agents may enhance antimicrobial effectiveness and prolong shelf life, but their chemical interactions remain unclear. Further studies are needed to clarify mechanisms of action, environmental influences, application methods in foods, and factors like safety, toxicity, nutrition, regulatory status, and consumer acceptance [137].

Several mechanisms may be involved in the antimicrobial effects of phenolic compounds, including cell lysis and/or changes in membrane permeability, enzyme inactivation, disruption of intracellular functions, and induction of cell death [123, 127]. These varieties of mechanisms are related to the structural diversity of phenolic compounds, especially when mixtures of different types are used [127]. Differences in efficiency have been reported for *E. coli* and *B. cereus*, with compounds such as curcumin, resveratrol, and cinnamaldehyde showing greater effectiveness than p‐coumaric acid and coniferyl alcohol [138].

Overall, Gram‐negative bacteria tend to be more resistant to the activity of phenolic compounds compared to Gram‐positive [128, 138, 139]. One example of this greater resistance was observed with curcumin: 240 µg/mL of the compound reduced *E. coli* populations by 2.84 log_10_ CFU/mL compared to results for *B. cereus*, where reduction was by 4.32 log_10_ CFU/mL after 48 h [138]. Similar differences were also reported with blueberry extracts, where MIC and MBC values were consistently lower for *L. monocytogenes* than for *Salmonella* Enteritidis [129]. Pomegranate peel extracts were also less effective against *Salmonella bongori* and *E. coli* compared to those recorded against *S. aureus* and *L. monocytogenes* [128]. The higher resistance of Gram‐negative bacteria to phenolic compounds may be due to the presence of an outer membrane that hinders diffusion of the compounds [128, 138, 139]. Moreover, the activity of efflux pumps in Gram‐negative bacteria, such as *Salmonella* Enteritidis, also seems to contribute to this resistance [129].

The efficiency of phenolic compounds is also influenced by their solubility, which affects their diffusion in the food matrix and thus their antimicrobial activity [127]. Additionally, even materials derived from the same plant species can vary in their bioactive compound content due to genetic factors, differences in maturity stages, influence of edaphoclimatic conditions, and extraction methods, among others [128]. The use of phenolic compounds as preservatives aims to reduce or even to replace synthetic additives, either alone or in combination with other antimicrobials [122]. Combined use is especially valuable because it may reduce the risk of microorganisms developing resistance [102, 122]. However, resistance development has been observed in *B. subtilis, B. cereus, Clostridium perfringens*, and *Clostridium sporogenes* exposed to thymoquinone after 24 h of incubation [140].

Another notable advantage of plant extracts is the resistance capability of lactic acid bacteria (LAB). For instance, pomegranate peel extracts exhibited antimicrobial activity against several foodborne pathogens such as *P. aeruginosa, B. cereus, S. aureus*, and *L. monocytogenes*, but not against *Lactiplantibacillus plantarum* and *Bifidobacterium animalis*, which may favor their use in fermented foods and the preservation of health benefits associated with LAB and bifidobacteria consumption [130]. Salim et al. [128] also reported lower inhibitory effects of pomegranate peel extracts against *Lacticaseibacillus casei* Shirota and *Limosilactobacillus reuteri*, while [141] found similar results in probiotic dairy beverages, further supporting the advantageous characteristic of phenolic compounds in preserving LAB.

**FIGURE 5:**
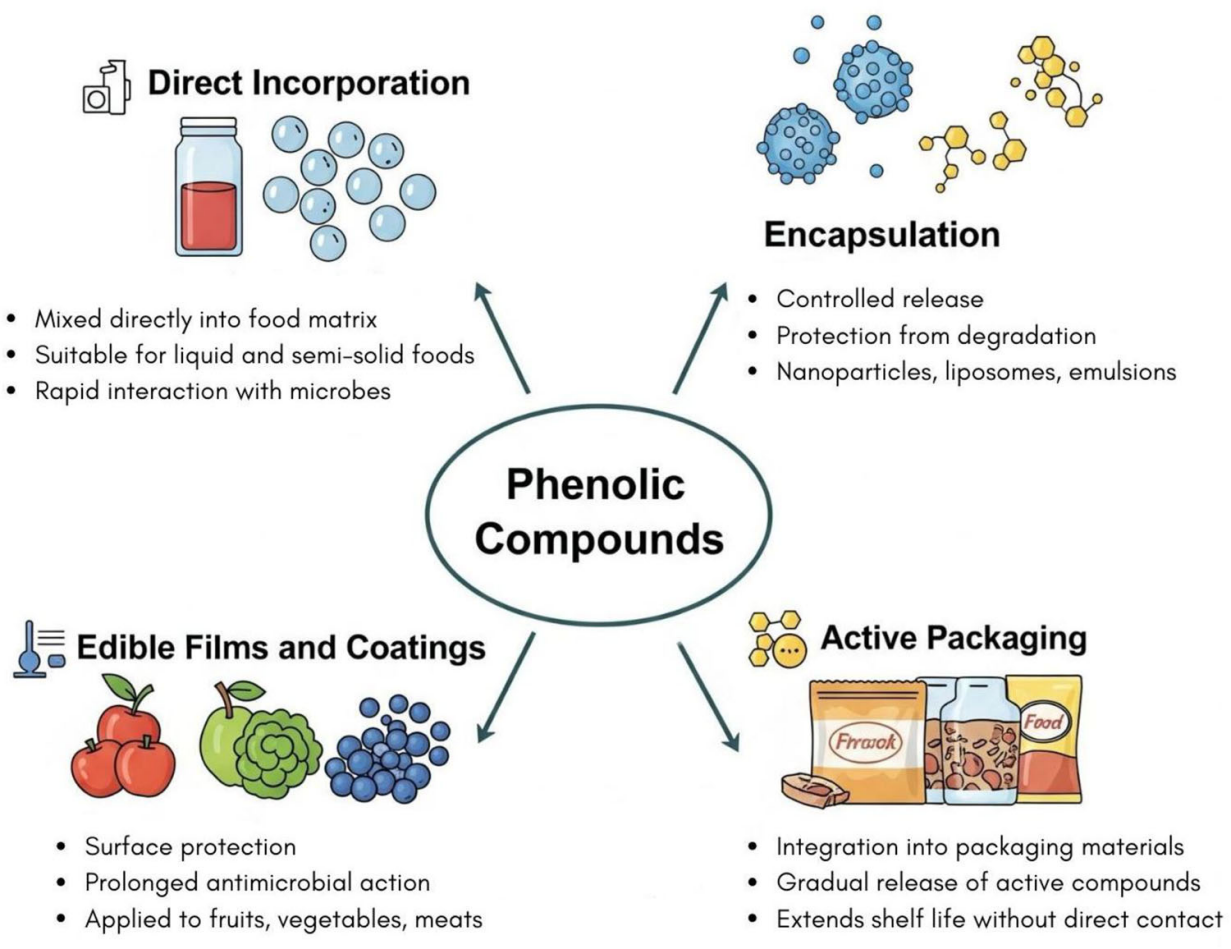
Strategies for using phenolic compounds in food preservation. Phenolic compounds can be used to extend the shelf life and improve the microbiological safety of the products through direct incorporation, in edible films and coatings, encapsulation, and as a packaging additive. Illustration created using AI‐assisted design tools.

The possible applications of phenolic compounds as food preservatives are diverse, including direct incorporation, encapsulation, use in films or coatings, and in packaging (Figure 5). Direct incorporation of the compounds by mixing with food ingredients is one of the simplest methods, but it is limited to the surface in the case of solid foods [102]. In this condition, the lack of contact with the target microorganism and interaction with food components may reduce the effectiveness of phenolic compounds [102, 122, 142].

The efficiency of these approaches has already been evaluated in peaches with soybean extracts, where a reduction in fungal growth on the fruit was observed without negative effects on quality parameters such as firmness, color, and acidity, suggesting the potential use of plant extracts as a postharvest technology, especially for fruits [143]. Promising positive results were observed in pork meat, where the addition of 200–300 mg GAE/kg olive leaf extracts minimized the production of conjugated dienes, hydroperoxides, and malondialdehyde, thereby reducing lipid oxidation and contributing positively to the product's acceptability [144]. Lower thiobarbituric acid reactive substances (TBARS) content was also observed with the addition of 0.2% of a plant extract mixture (*Cymbopogon citratus, Curcuma longa* L., *Myristica fragrans, Zingiber officinale*, and *Thymus vulgaris)* in raw rabbit meat [145],0.2% Flos sPinilla et al.ophorae in Chinese sausages [146], and pitomba extracts in fresh pork sausage [147]. These examples demonstrate the feasibility of using extracts rich in phenolic compounds to improve the preservation of meat products.

In addition to reducing lipid oxidation, supplementation with plant extracts also can impact microbial load, as shown by Ahmad et al. [134], where the addition of 1% coriander extract reduced both lipid oxidation and viable aerobic bacterial plate count in poultry meat patties. Cheah and Abu Hasim [148] also observed lower oxidation and microbial load after cooking in minced meat added with 10% galangal (*Alpinia galanga*) extract. Similarly, Elhadef et al. [149] observed lower amounts of aerobic mesophiles, psychrotrophs, and Enterobacteriaceae in raw chicken meat supplemented with palm seeds ethanolic extract. Shoqairan et al. [150] reported lower aerobic mesophile counts in beef burgers prepared with cinnamon powder. However, Hettiarachchy et al. [151] found no changes in the load of psychrotrophic microorganisms in beef patties added with 500–1000 ppm of fenugreek (*Trigonella foenum‐graecum*) extracts. These findings suggest the potential beneficial applications for phenolic compounds as food preservatives. However, the industrial application of these compounds is not a panacea and is still limited by their low stability, especially due to oxidation, requiring strategies to minimize these possible disadvantages [122, 152]. In this context, the encapsulation process—by spray drying, emulsification, solvent precipitation, electrospinning, or liposomes—can be applied to produce micro‐ or nanocapsules to maximize the bioavailability and stability of phenolic compounds (Figure 5) [102].

Generally, nanomaterials tend to offer greater surface area, stability, and resistance, which contribute to lower susceptibility to changes in pH and temperature [127]. Phenolic compounds with hydrophobic characteristics are particularly benefited by encapsulation, as they promote better distribution in the food matrix and minimizes its concentration in the lipid portion [102]. Different materials such as alginate, starch, gelatin, xanthan gum, and carrageenan can be used to encapsulate hydrophilic compounds, while hydrophobic compounds are converted into liposomes or micelles with the aid of phospholipids [102, 153].

The use of the mentioned encapsulation strategy for food preservation has already been explored by Sirini et al. [154], who developed beef patties with kiwifruit peel extract encapsulated in maltodextrin by spray drying and reported reduced lipid oxidation with the use of 2.5 and 5.0 g/kg during storage, although without impact on viable aerobic bacterial plate count, LAB, or *Pseudomonas* spp. The absence of antimicrobial activity of the extract was attributed to the use of milder extraction conditions, which may have resulted in lower levels of bioactive compounds [154]. Matencio et al. [155] studied the effect of oxyresveratrol encapsulated in cyclodextrin by spray drying added to orange juice and milk and observed increased antioxidant capacity of the products and a bacteriostatic effect against *E. coli*. Similarly, Pinilla et al. [156] evaluated the use of garlic extract encapsulated in liposomes and observed inhibition halos against all tested fungi (*Penicillium expansum, Aspergillus niger, Penicillium herquei, Fusarium graminearum*, and *Aspergillus flavus*) and recorded longer shelf life in breads supplemented with the explored compound. Sharayei et al. [157] also demonstrated an extended shelf life in cupcakes added with pomegranate peel extract encapsulated in maltodextrin, in addition to better sensory acceptability of the products supplemented with the extract (free or encapsulated).

These data suggest that encapsulated phenolic compounds generally contribute to improved product safety. However, the success of the strategy depends on several factors, such as the compound dosage used, the materials employed for encapsulation, particle size, and distribution in the food, among others [102, 158]. Another important issue is toxicity assessment, requiring more in vivo studies that evaluate the toxicity of both free and encapsulated compounds, as well as their impact on gut microbiota, whose effects are still poorly understood [159].

Another important approach for applying phenolic compounds as food preservatives is through their incorporation into edible films and coatings applied in direct contact with the surface of the food (Figure 5) [102]. The presence of these edible films and coatings represents a barrier to gases, temperature, ultraviolet light, moisture, and microorganisms, in addition to reducing the oxidation of food ingredients, which increases shelf life [160, 161, 162]. Usually, coatings are applied or formed directly on the surface of the food by dipping, spraying, or spreading the coating solution, while films are sheets used to package the food [160, 161]. Different materials can be used to produce coating films and edible coatings, but the use of agricultural residues has gained prominence for minimizing competition with food resources, in addition to having lower cost and environmental impact. The compounds used to produce edible films and coatings must be safe and carefully chosen, since, in addition to considering the desirable functional properties of the coatings, they must comply with the regulations of each country [160, 161, 162]. Moreover, if allergenic products such as soy, milk, or wheat are used, for example, this must be clearly indicated to the consumer [160].

The incorporation of phenolic compounds into coating films can improve their functionality and contribute to improving food quality and safety [162]. One example was observed in pork packaging, where the presence of pomegranate peel extract in the film wrapping the product increased its shelf life by 2–3 days at 4°C by reducing microbial growth and chemical spoilage [163]. Films made with a chitosan and polyvinyl alcohol blend also showed greater inhibition of the growth of strains belonging to species *E. coli, P. aeruginosa, S. aureus, B. subtilis*, and *C. albicans* when incorporated with *H. sabdariffa* extract, and the effect was directly proportional to the concentration of the extract [164]. The presence of sea fennel (*Crithmum maritimum* L.) extract in sodium alginate—chitosan films also favored the preservation of strawberries due to lower microbial growth, softening, and weight loss of the fruit [135]. Coating films with incorporated olive leaf extract also induced lower lipid oxidation of cooked meat refrigerated for 14 days without affecting product quality parameters, suggesting potential use in meat products [165]. However, there are still several challenges in the production and use of edible films and coatings, such as regulatory issues, consumer skepticism, large‐scale production, higher production cost, and the possibility of sensory alterations of the product, especially in texture and flavor [162].

Antimicrobial compounds such as phenolic compounds can also be incorporated into active packaging to extend food preservation (Figure 5). Among the most common techniques are the addition of the compound in the headspace, its inclusion in sachets that allow diffusion into the headspace, and incorporation into polymers [102, 123, 142]. The use of bioactive polymers has gained prominence due to the possibility of replacing and/or reducing the use of petroleum‐derived plastic packaging, which decreases the environmental impact of the sector [166]. The efficacy of this strategy has already been evaluated by Ferreira et al. [167], who developed poly (butylene adipate‐co‐terephthalate) films with nanocapsules containing cinnamon essential oil and observed that films with 8% improved tensile strength and showed higher melt fusion values, in addition to exhibiting antimicrobial activity against *E. coli*, suggesting the potential use of these films as active packaging. In addition, Nahas et al. [168]. developed carboxymethyl cellulose films containing phenolic compounds from rosemary wastewater and reported that films with aqueous extracts improved UV light barrier and increased yellowing index, in addition to exhibiting antioxidant activity (300% FRAP and 700% TEAC) higher than the control, suggesting potential use for light‐sensitive food products. Films with rosemary wastewater essential oil also presented benefits such as greater flexibility, an interesting characteristic for use in flexible wraps [168].

Studies in food matrices have also demonstrated the potential of this strategy, such as that by Colin‐Chavez et al. [169], who developed sachets with oregano oil microencapsulated by spray drying and observed that adding the oil at concentrations of 0.15, 0.25, and 1 g inhibited the in vitro growth of *Colletotrichum gloeosporioides, Colletotrichum acutatum, Diaporthe passiflorae*, and *Neoscytalidium hyalinum*. A similar effect was also observed in avocados using the sachets in a sealed humidified chamber, where the addition of 0.15 g of oil reduced the injury caused by *C. gloeosporioides*, without affecting the firmness and color of the fruit, suggesting the potential of the product as a postharvest technology [169]. Sachets with *Zataria multiflora* essential oil loaded in halloysite nanotubes (2:1, 20%) reduced the *E. coli* O157:H7 population in cheese by 1.6 log CFU/g, without showing cytotoxicity to normal human dermal fibroblasts [170]. Adverse effects from incorporating phenolic compounds have also been observed, as in the study by Malherbi et al. [171], which showed that the addition of *guabiroba* (*Campomanesia xanthocarpa* L.) pulp in blend films for sachets in olive oil preservation increased the fragility of the films and did not enhance the oxidative stability of the product.

Another possible application of phenolic compounds in food matrices is through incorporation into polymers that can be applied to different types of products, such as fruits, meats, cheeses, and oils, among others. Using this strategy, Acquavia et al. [172] demonstrated that a polylactide‐based bioplastic incorporated with tea waste compounds showed higher UV light‐blocking capacity and ductility, as well as higher antioxidant capacity and antimicrobial properties against *E. coli*. The polymers were tested in pear preservation, and lower moisture loss, higher antioxidant capacity, and less enzymatic browning of the product were observed, similar to the product packaged with low‐density polyethylene film. Martiny et al. [173] developed a carrageenan‐based film with olive leaf extract and observed that the addition of the extract increased thickness and altered the film's color, in addition to reducing psychrotrophic microbial growth in lamb meat by approximately five times. Andrade et al. [174] suggested the application of polylactic acid polymers with rosemary extract and observed lower lipid oxidation in beef meat and lower aerobic mesophile populations, with the antimicrobial effect noted after the first few days of storage. Freitas et al. [175] also developed polylactic acid polymers, but incorporated extracts from grape stems obtained through subcritical water extraction and showed that the addition of the extracts increased UV protection, altered the polymer's coloration, and minimized the oxidation of sunflower oil. Meanwhile, Cui et al. [176] developed a nanofibrous membrane of polyethylene oxide containing liposomes with eugenol adsorbed on silica and observed stable antioxidant activity over 60 days of storage, in addition to lower lipid oxidation in beef and better sensory acceptability of the product.

In general, most antimicrobial potential assays of phenolic compounds are usually conducted in vitro, under ideal conditions for the target microorganism. However, the efficiency of these compounds in situ in food matrices may differ significantly, as food components—especially proteins and lipids—can interact with phenolic compounds and markedly reduce their antimicrobial activity. Therefore, it is required to encourage the development of assays under the conditions in which the compound is intended to be applied [102, 122, 127]. Another important point to consider is the sensory characteristics of phenolic compounds, such as color and taste, which can adversely affect food sensory attributes and lead to consumer rejection [123, 127].

### 7.2 Functional Foods and Nutraceuticals

The addition of phenolic compounds to foods not only contributes to their preservation but can also enhance the nutritional quality of the product, thereby positively influencing its consumption due to consumers' growing interest in more natural and healthier food choices (Figure 4) [118, 177]. Phenolic compounds are associated with the prevention of chronic and degenerative diseases, thus their addition to foods promotes a functional aspect of the product [177, 178].

Various food products, such as dairy, bakery or meat products, can be enriched by phenolic compounds, with positive effects on increasing their antioxidant content [118, 179, 180, 181]. Usually, the enrichment process is carried out by adding ingredients and/or extracts rich in phenolic compounds to the product's ingredients [118, 182]. However, encapsulation techniques can also be employed to improve the stability and bioavailability of phenolic compounds [177].

The benefits from fortification with phenolic compounds on antioxidant activity were demonstrated by Pedziwiatr et al. [182], who evaluated the fortification of cookies with extracts from different plants and observed an increase of over 300% in phenolic content. This led to enhanced antioxidant activity as measured by ABTS0+, FRAP, and ORAC assays, and to the inhibition of α‐amylase and α‐glucosidase enzymes in fortified cookies, especially those made with chokeberry and haskap berry. Kral et al. [183] also reported an increase in the antioxidant activity of cookies enriched with herbs or grape seed flour, which was directly proportional to the amount of ingredient used.

Similarly, Saggin et al. [184] found increased antioxidant activity in muffins made with 5%–10% flour from Brazilian guava (*Myrcia oblongata* DC.), a native Brazilian fruit. Botta‐Arias et al. [185] developed orange peel snacks with plant extracts that enhanced antioxidant activity, especially when prickly pear was used. Lopez‐Parra et al. [181] showed increased phenolic content and antioxidant activity in lamb burgers enriched with cherries. These findings suggest the potential for enriching various types of foods with phenolic compounds. The health benefits of enriched foods have been previously reported by Macho‐Gonzalez et al. [186], who demonstrated metabolic improvements, such as reduced insulin and glucose levels (*p*<0.001) and enhanced antioxidant defense in the proximal mucosa of Wistar rats fed meat enriched with carob fruit extract.

However, since phenolic compounds can influence the sensory characteristics of products, it is essential to conduct sensory evaluations to assess consumer acceptance of the desired compound concentrations [118]. In the development of fortified dairy products, Kandyliari et al. [118] observed that kefir, cream cheese, yogurt, and vegan yogurt‐like products enriched with herbs or plant by‐product extracts had lower acceptability than their unenriched counterparts. Similarly, Kral et al. [183] showed that cookies enriched with higher herb concentrations were not well accepted, highlighting the importance of balancing functionality and sensory acceptance.

Another point to consider is the ability of phenolic compounds to interact with the food matrix and undergo alterations during processing, which can change their structural characteristics. Thus, the enrichment process must be carefully optimized to avoid undesirable changes in texture, odor, and/or flavor characteristics [180]. Changes in the texture of fortified products have been previously demonstrated in biscuits enriched with spices and herbs, showing a significant reduction in the hardness and friability of the enriched products [183].

In addition to their potential for food fortification, phenolic compounds can also be used as dietary supplements and nutraceuticals [187]. Increased antioxidant activity in different organs, such as the kidney and liver, reduction of lipid peroxidation, and mild hypoglycemic and hypolipidemic effects have already been demonstrated in Wistar rats using an extract from chestnut shells, reinforcing the potential use of these compounds in the prevention of metabolic diseases [187].

### 7.3 Flavor and Color Enhancement

Color is a key sensory attribute influencing consumer acceptability of food products. Many phenolic compounds, including flavonols, anthocyanins, and their oxidation products, act as pigments, and their addition to foods may affect the color of the final product either positively or negatively (Figure 4) [188]. Kral et al. [183] demonstrated this impact by showing that biscuits enriched with cinnamon, mint, cloves, or grape seed flour exhibited a darker coloration. Botta‐Arias et al. [185] also showed that the addition of plant extracts to orange peel‐based snacks significantly affected (*p* < 0.05) the product's color, although without impacting consumer acceptability. In contrast, the enrichment of dairy products with extracts from different herbs did not result in noticeable sensory changes to consumers [118].

On the other hand, Pedziwiatr et al. [182] reported a positive effect from the addition of phenolic compounds in cookies fortified with extracts from various plants. In this case, anthocyanins, polymeric procyanidins, and flavonols had the most positive impact on consumer acceptability. Similarly, Tang et al. [146] demonstrated an improvement in the aroma (*p* < 0.05) of Chinese sausages enriched with *Flos sophorae*, and Elhadef et al. [149] observed better sensory attributes—including color, appearance, odor, and overall acceptability—during the storage of raw chicken meat treated with palm seed extract. In summary, the addition of phenolic compounds to food products may induce changes in their sensory properties, and the effects on consumer acceptance must be carefully assessed.

### 7.4 Cosmetical and Personal Care Products

The growing demand for natural substances in cosmetics and personal care products has led to the inclusion of an increasing number of natural ingredients in these formulations [167]. In this context, phenolic compounds have gained prominence due to their biological properties, which may contribute to anti‐photoaging and photoprotective properties, supporting the development of cosmeceutical products (Figure 4) [167, 189].

Although the mechanism of action of phenolic compounds is not yet completely elucidated, it is known to be related to their antioxidant activity, their capacity to absorb ultraviolet (UV) light, and/or their ability to modulate inflammatory pathways [124, 190]. From this perspective, materials from species such as *Vitis vinifera, Glycine soja, Acacia decurrens, Glycyrrhiza glabra, Limnanthes alba, Theobroma cacao, Calendula officinalis, Helianthus annuus, Simmondsia chinensis*, and *Butyrospermum parkii* are frequently used in cosmetic products in Europe [167]. Such materials provide compounds like anthocyanins, flavonols, tannins, stilbenes, and hydroxycinnamic acid derivatives, which contribute to product efficacy [167].

The plant part used, and the extraction method may vary, although extracts and oils are the most commonly employed forms [167]. The limited knowledge about the metabolites produced and the diversity of plant species indicates there is still great potential to be explored, as highlighted by Kukula‐Koch et al. [191], who described the cosmetic potential of several species from the genus *Vaccinium*, due to their rich composition of phenolic acids, flavonoids, flavonoid glycosides, anthocyanins, and complex tannins. *Foeniculum vulgare* Mill. also stands out for its versatility, being used in the form of leaf extracts, fruit powders, seeds, and oils [192]. In this sense, the inclusion of phenolic compounds in cosmetics may help mitigate the effects of skin aging by inhibiting enzymes such as collagenase and elastase, as demonstrated by Bose et al. [193], who evaluated extracts of the orchid *Malaxis acuminata*, and by Moreira et al. [194], who observed reductions of 25% and 45% in collagenase and elastase activity, respectively, using 100 µg/mL of *Eugenia dysenterica* leaf extract. Nema et al. [195] also reported anti‐hyaluronidase and anti‐elastase activity in lyophilized juice of *Cucumis sativus* fruit. Anti‐elastase and anti‐collagenase activities were also detected in extracts from nine and sixteen different plants, respectively, as evaluated by Thring et al. [196].

Beyond their inhibitory effects on aging‐related enzymes, phenolic compounds may act in sun protection due to their ability to absorb UV radiation, comparable to that of synthetic sunscreen substances. This activity has been demonstrated by several authors, including Bose et al. [193] with *M. acuminata* extracts, Lima et al. [197] with babassu mesocarp flour extracts, and Nunes et al. [198] using extracts of *Luehea paniculata, Lippia microphylla, Dimophandra gardneriana*, and *Amburana cearenses*.

In addition to photoprotection, phenolic compounds in plant extracts can also modulate the proliferative response to UVB, as observed by Petrova et al. [199] with topical use of honeybush (*Cyclopia* spp.) extracts. Their use reduced sunburn signs, edema, epidermal hyperplasia, and cell proliferation (as measured by ornithine decarboxylase expression), as well as DNA damage, evidenced by reduced levels of GADD45 and OGG1/2, suggesting potential in reducing inflammatory skin conditions. Interestingly, the individual application of the main extract components (hesperidin and mangiferin) was less effective than the crude extracts, indicating the relevance of other co‐occurring compounds [199]. Batista et al. [200] also demonstrated the feasibility of using hydroalcoholic extract of red propolis in hydrogels, producing stable formulations with suitable application and skin retention properties, as well as skin compatibility in humans.

Although plant‐derived phenolic compounds show great potential for cosmetic applications, their use is often limited by the lack of evidence on toxicity, safety, and allergenic potential [192]. Other challenges include large/industrial scale production of the plant material and further extraction techniques, which may directly affect the yield of the target compounds, and the stability and bioavailability of phenolic compounds [167].

### 7.5 Pharmaceuticals

The antioxidant activity of phenolic compounds suggests their potential use in the treatment of various diseases, such as cancer, diabetes, hypertension, infections, cardiovascular diseases, among others (Figure 4) [125, 126]. In addition to their ability to eliminate reactive oxygen species (ROS) and chelate metal ions, phenolic compounds also inhibit lipid peroxidation and stimulate antioxidant enzyme activity, which directly impacts oxidative stress—a key factor in the pathogenesis of many diseases [201].

Therefore, the dietary intake of antioxidants has already been associated with lower levels of oxidative damage [125]. Moreover, several phenolic compounds have been indicated as therapeutic alternatives for the treatment of diseases—for example, gallic acid for diabetes and cancer [202, 203], resveratrol for cancer [204], hydroxymatairesinol as a neuroprotective agent [205], and sesamin for metabolic syndrome [206], among others.

As with other applications, challenges remain to be addressed, such as low bioavailability, chemical instability, and limited understanding of their mechanisms of action and hepatic metabolism. Additionally, further robust evidence is needed regarding appropriate dosage, adverse effects, and potential drug interactions. From this perspective, phenolic compounds may significantly contribute to the treatment of various diseases, especially those in which oxidative stress plays a central role in their pathogenesis [207].

## 8 Advanced Applications of Phenolic Compounds: Regulatory Landscape and Bioeconomic Opportunities

The growing knowledge regarding the consumption of phenolic compounds and their potential health benefits has increased interest in their use across various nutritional and cosmetic products. However, the inclusion of health claims on product labels necessitates compliance with complex regulations established by health authorities in each country or region, such as the European Food Safety Authority (EFSA) in the EU, the China Food Additives and Ingredients Association in China, the Food and Drug Administration (FDA) in the USA, and the National Health Surveillance Agency (ANVISA) in Brazil [102].

Approval of the use of health claims requires robust scientific evidence demonstrating the suggested benefits, conditions of use, necessary dosages, and the safety and toxicity of the compound. This is a complex process due to the stringent requirements of regulatory agencies, which demand clinical studies that adequately substantiate the desired claims for the product [102, 122, 208]. As a result, the number of officially approved health claims remains limited. One example of an approved claim is the protection of lipids from oxidative stress, which can only be used on olive oils providing at least 5 mg of hydroxytyrosol per 20 g of olive oil [209].

Numerous applications for health claims are rejected by health authorities due to insufficient evidence linking the product's consumption to the proposed benefit. One such example is the application for Pacran, a cranberry powder, which sought to claim a protective effect against pathogenic bacteria in the urinary tract [210]. Another was the application for Joselito ham, which proposed a claim related to low‐density lipoprotein (LDL) reduction and coronary heart disease. This was denied due to the failure to establish a clear relationship between the product's consumption and reductions in blood pressure or LDL cholesterol [210].

These data underscore the rigorous evaluation undertaken for the approval of health claims and reflect the necessary diligence in product labeling, especially for food items [208]. Such careful product labeling is crucial to provide consumers with opportunities for more conscious and better‐informed food choices, thereby contributing to public health [122]. Advances in knowledge on this topic, extraction techniques, and clinical trials will likely expand the availability of foods with functional properties, thereby facilitating consumer access to these products.

On the other hand, food waste remains a global issue, leading to the disposal of approximately one‐third of all food produced for human consumption [211, 212]. These losses occur throughout the entire production chain and have significant environmental impacts, due to the unnecessary use of natural resources, such as land and water, as well as social consequences by exacerbating food insecurity [211, 213].

In this context, strategies that promote the valorization of food waste have gained prominence by fostering circular economy practices and minimizing environmental impacts [104]. One such strategy involves the use of food by‐products as sources of bioactive compounds, particularly phenolic compounds [213]. Various food residues (including peels, seeds, and fruit pomace) have been identified as promising sources of phenolic compounds using pomegranate peel extracts [116, 141], grape seed flour [118], *jabuticaba* (*Myrciaria jaboticaba*) peel [117] and using orange and tomato peels [115]. These alternatives are essential for ensuring the sustainability of production systems by enabling the development of natural products while reducing dependence on natural and fossil resources and may also provide economic benefits [115].

## 9 Mechanisms of Action

### 9.1 Disruption of the Cytoplasmic Membrane of Microorganisms

Phenolic compounds exert antimicrobial activity through several mechanisms, including direct interaction with the bacterial cell membrane, leading to structural compromise [214]. Even in bacteria possessing complex and multilayered structural barriers, these phenolic compounds can penetrate the bacterial defenses and cause significant damage, such as membrane destabilization and inhibition of essential metabolic processes [215]. The membrane structure not only protects bacteria from adverse environmental conditions, but it is also essential for nutrient exchange and metabolite secretion [216]. Gram‐positive bacteria exhibit greater susceptibility to phenolic compounds, mainly due to the absence of an outer membrane and the exposed peptidoglycan‐based cell wall [217]. Functional groups present on the bacterial cell surface interact with polyphenols, enabling, for example, the hydroxyl groups of these compounds to break the bonds between peptidoglycans, thereby compromising the cell wall [218]. The effectiveness of this type of action depends both on the structural nature of the phenolic compounds and the physiological profile of the bacterium [219].

In contrast, Gram‐negative bacteria possess a more complex cellular architecture, consisting of an outer membrane rich in phospholipids and lipopolysaccharide (LPS), an intermediate peptidoglycan layer, and an inner cytoplasmic membrane [220]. This composition makes these organisms relatively more resistant to the antimicrobial effects of phenolic compounds [221]. In these cases, the mechanism of action involves the accumulation of hydroxyl groups within the lipid bilayers, which disrupts the interactions between lipoproteins and increases membrane permeability. This process can ultimately lead to membrane rupture, loss of cell morphology, metabolic disruption, and leakage of essential intracellular components. The destabilization of the phospholipid bilayer, therefore, compromises cell viability by interrupting physiological processes and cell division [222, 223, 224]. Beyond their action on membranes, phenolic compounds can also directly interfere with the activity of bacterial enzymes.

### 9.2 Inhibition of Enzymatic Activity

The remarkable ability to inhibit the activity of several essential bacterial enzymes is a characteristic of several polyphenols, which modulate critical metabolic functions vital for microbial viability and pathogenicity [225]. These compounds interact with enzymes through covalent and non‐covalent bonds, influenced by factors such as conformation, molecular weight, hydrophobicity, and affinity for functional groups, particularly sulfhydryl groups and metal ions whose chelation compromises catalytic reactions essential for bacterial survival [226, 227]. Compounds such as theaflavins, condensed tannins, and flavonoids have demonstrated strong inhibition of key enzymes, including NADH dehydrogenases, c‐di‐AMP synthases, and dihydrofolate reductase, directly affecting energy metabolism, cell signaling, and bacterial replication [228, 229]. Additionally, phenolic compounds inactivate enzymes associated with bacterial adhesion, such as glycosyltransferases, and irreversibly modify enzymes located in the membrane, such as glucan synthase, through covalent reactions with oxidized products [223, 230]. Furthermore, it has been shown that these compounds can even neutralize important bacterial toxins, including *E. coli* enterotoxins, the cholera toxin of *Vibrio cholerae*, and the exotoxin A of *P. aeruginosa*, by inhibiting their catalytic activity or preventing their binding to cellular receptors [231]. This broad inhibitory action results in metabolic deregulation, loss of cell integrity, and impairment of pathogenicity, highlighting the potential of polyphenols as broad‐spectrum natural antimicrobial agents [225, 228, 231].

### 9.3 Oxidative Stress Induction

The induction of physiological stresses, particularly oxidative stress, represents a fundamental antimicrobial mechanism associated with various phenolic compounds, reflecting a significant aspect of their bioactive properties [218, 232, 233]. Although the bacterial responses to endogenous oxidative stress which are triggered by reactive oxygen species such as superoxide anion (O_2_^−^) and hydrogen peroxide (H_2_O_2_), and lead to damage of metalloenzymes and DNA, are well characterized, recent studies have expanded our understanding of how exogenous phenolic compounds can interfere with these mechanisms. These interactions can either exacerbate oxidative damage or modulate alternative cellular stress response pathways [232, 234]. Recent investigations have further elucidated how phenolic compounds interact with these mechanisms, potentially intensifying them or triggering alternative cellular responses. For example, Lima et al. [235] demonstrated that combinations of specific phenolic compounds induced morphological changes, such as cell elongation in *P. aeruginosa*, suggesting stress response, in addition to acting synergistically with antibiotics to eradicate biofilms of this organism.

The understanding of how phenolic compounds trigger the production of reactive oxygen species has been significantly deepened through studies utilizing omics and molecular biology approaches [236, 237]. Recent research has revealed that certain compounds can directly interact with components of the bacterial electron transport chain [238, 239] or with cytoplasmic flavoproteins [240, 241], diverting electrons to molecular oxygen and resulting in a robust production of superoxide [242]. Subsequently, the dismutation of superoxide generates hydrogen peroxide, which can diffuse and oxidize sensitive targets or, in the presence of transition metal ions such as iron (Fe^2^^+^), be converted into the highly reactive hydroxyl radical (•OH) through Fenton‐type reactions, a process exacerbated by phenolic compounds with chelating and redox‐active capacity. This oxidative cascade overwhelms the bacterial antioxidant defense systems, such as superoxide dismutase (SOD), catalase, and the glutathione reductase system, leading to lipid peroxidation of membranes, carbonylation of essential proteins (including those with vulnerable iron‐sulfur clusters), and extensive DNA damage, ultimately resulting in cell cycle arrest, mutagenesis, or cell death [242, 243, 244].

Moreover, phenolic compounds have demonstrated the ability to interfere with global regulatory systems, such as *quorum sensing* (QS), a cell communication mechanism that coordinates gene expression in response to bacterial population density [245]. This interference can occur through the inhibition of the production, release, or detection of signaling molecules, also called autoinducers, thereby modulating critical physiological processes, including redox homeostasis and the response to oxidative stress [246]. The effectiveness of this modulation is often determined by various factors, such as the specific chemical structure of the compounds, their concentrations, the physiological state of the bacteria, and microenvironmental conditions, including pH, the presence of metal cofactors, and the degree of environmental stress. All these factors can directly influence the capacity of the phenolic compounds to disrupt cellular signaling and induce redox imbalance [247].

### 9.4 Interference With Microbial Signaling (*Quorum Sensing*)

The growing antimicrobial resistance has driven the search for alternative strategies aimed at attenuating bacterial virulence without exerting selective pressure on microorganisms [248]. Interference in the bacterial QS system by phenolic compounds has emerged as a promising approach [246]. Phenolic compounds such as curcumin and resveratrol have demonstrated the ability to directly interfere with QS systems [249, 250, 251], resulting in the inhibition of violacein production by *Chromobacterium violaceum* [252]. These same compounds also promote reduced biofilm formation and impair motility in various pathogenic bacteria, reinforcing their applicability as antivirulence agents with therapeutic or biotechnological potential [245, 251].

Other phenolic compounds, such as baicalein, rosmarinic acid, quercetin, and gallic acid, have also stood out for their ability to inhibit multiple QS‐controlled phenotypes associated with the virulence of *P. aeruginosa*, including the production of pyocyanin, biosurfactants, hydrolytic enzymes, and motility. Baicalein and rosmarinic acid were particularly effective in suppressing swarming motility and pyocyanin production, even at concentrations that did not affect bacterial growth. Quercetin was active in reducing rhamnolipids and proteases, while gallic acid showed consistent and broad antivirulence effects [253].

Additionally, the phenolic extract from the pulp of *Eugenia uniflora* L. (surinam cherry) fruits demonstrated significant antivirulence activity, reducing biofilm formation by *Serratia liquefaciens* on polystyrene surfaces even at sub‐inhibitory concentrations. This reduction resulted in lower polysaccharide, protein, and extracellular DNA content in the biofilm matrix [254]. The synergistic effect between the phenolic extracts and the furanone C30 highlighted the potential of these natural compounds in mitigating bacterial virulence through QS‐related mechanisms [254]. Complementarily, phenolic extracts from onion (*Allium cepa*), rich in quercetin, particularly in its aglycone form, also demonstrated the ability to modulate QS systems in various Gram‐negative bacteria [255]. In *C. violaceum*, significant inhibition of violacein production was observed, while in *P. aeruginosa* and *Serratia marcescens*, both the extracts and isolated forms of quercetin reduced swarming motility. In silico studies further reinforced these findings by showing a strong affinity of quercetin aglycone for the autoinducer‐binding domains of the regulatory proteins CviR and LasR, suggesting that its antivirulence activity may occur through competitive blocking of these QS receptors [255]. Although studies on the biological effects of polyphenols are promising, understanding how their structural properties relate to antimicrobial activity has become a strategic need for the rational advancement in the development of new bioactive compounds.

### 9.5 Antibacterial Properties of Polyphenols: Characterization and Quantitative Structure–Activity Relationship

Recently, approaches based on quantitative structure–activity relationship (QSAR) have been employed to rationalize and predict the bioactivity of polyphenols against microorganisms. Highly predictive QSAR models developed by Bouarab‐Chibane et al. [256] have correlated molecular properties such as lipophilicity, electronic distribution, and molecular volume with the antibacterial activity of 35 polyphenols against strains like *E. coli*, *Salmonella* Enteritidis, and *S. aureus*. The models demonstrated strong predictive power (R^2^ > 0.85) and compliance with the criteria established by the Organization for Economic Co‐operation and Development (OECD), reinforcing the applicability of these algorithms in the design of new natural antibacterial agents. Another study by Araya‐Cloutier et al. [257] showed that structural modifications, such as glycosylation and the presence of hydroxyl groups at specific positions on the phenolic ring, significantly influence the bactericidal activity of polyphenols, particularly under altered metabolic conditions such as hyperglycemic states. Moreover, recent studies have explored the effects of prenylation in flavonoids, revealing that this structural modification can increase the permeability and antimicrobial activity of these compounds. QSAR models developed based on prenylflavonoids have demonstrated that the presence of prenyl groups is associated with a greater capacity to disrupt bacterial membranes, particularly in Gram‐positive bacteria [258]. These integrated approaches of phytochemical characterization and computational modeling represent an innovative path to develop more effective and sustainable antimicrobial strategies.

### 9.6 Antimicrobial Activity of Naturally Occurring Phenols and Derivatives Against Biofilm and Planktonic Bacteria

Bacteria can exist in two distinct physiological forms: as planktonic cells, which are free‐living in the environment, or as organized biofilms, which are complex structures attached to surfaces and surrounded by a self‐produced polymeric extracellular matrix [259, 260]. These structural and functional differences grant biofilms a remarkable tolerance to antimicrobial agents and environmental stress, making their eradication challenging in both clinical and industrial settings [261, 262]. While planktonic cells are more susceptible to the action of antimicrobial compounds, such as phenolics [263], biofilm‐associated cells exhibit significantly higher tolerance, requiring higher concentrations and prolonged exposure times for inactivation [264]. This resistance is related not only to the physical barrier imposed by the extracellular matrix [259] but also to the different cellular physiological states and differential expressions of genes associated with survival, cell‐to‐cell communication via QS, and persistence [265, 266].

Several natural phenolic compounds, such as eugenol, thymol, vanillin, and carvacrol, have demonstrated potent antimicrobial activity against planktonic bacteria, primarily through mechanisms that involve destabilization of the cell membrane and the loss of cytoplasmic content [267]. However, the effectiveness of these compounds against bacterial biofilms is considerably diminished, requiring higher concentrations to achieve comparable effects [268]. This reduction in efficacy is attributed to the complex extracellular matrix of biofilms, composed of polysaccharides, proteins, extracellular DNA (eDNA), and lipids, which serve as a significant physical barrier that can impede or delay the penetration of antimicrobial agents, including phenolic compounds, to bacterial cells within the biofilm [269]. Electrostatic or hydrophobic interactions between phenolics and matrix components can also contribute to the sequestration of these compounds [270]. Additionally, physiological heterogeneity within biofilms, with cells existing in various metabolic states, including dormancy or persistence in regions with limited nutrients and oxygen, renders bacteria less susceptible to antimicrobials that depend on metabolically active targets [271].

To overcome these challenges, innovative strategies have been investigated, including the chemical derivatization of phenols to optimize their physicochemical properties and enhance penetration into biofilms [272] as well as the combined use of phenolic compounds among themselves or with other antimicrobials [235, 253]. A promising example is the prodrug strategy, such as the use of acetoxymethyl (AM) iminodiacetate groups linked to phenols, which can facilitate entry into bacterial cells and subsequently be cleaved by intracellular esterases to release the active phenol, thereby increasing its local concentration and efficacy against biofilms [268]. Other approaches include the use of nanocarriers to encapsulate and deliver phenols to the biofilm [273] and the combination of phenols with other antimicrobial agents or biofilm dispersing agents to achieve synergistic effects [235, 274].

## 10 Preventing Microbial Infections With Natural Phenolic Compounds

Antimicrobial resistance represents one of the greatest challenges to global public health in the 21st century, significantly reducing the effectiveness of conventional antibiotics and compromising the treatment of common bacterial infections [275]. In this concerning context, natural bioactive compounds, particularly phenolic compounds derived from plants, fungi, and marine organisms, have attracted increasing interest due to their multifunctional antimicrobial properties [276]. The structure of these compounds, rich in hydroxyl groups and other polar or nonpolar functions, enables a variety of mechanisms of action against pathogenic microorganisms [277]. As highlighted by Lobiuc et al. [223], these compounds can interfere with vital bacterial cell processes, including membrane integrity, protein synthesis, energy metabolism, and DNA integrity.

One of the main advantages of phenolic compounds lies in their ability to act through multiple mechanisms of action, which significantly reduces the risk of resistance selection [275]. The synergistic interaction between phenolic compounds and conventional antibiotics has been widely documented as a promising strategy to restore antimicrobial efficacy against multidrug‐resistant strains. Recent studies have shown that flavonoids such as rutin and quercetin enhance the effects of antibiotics like gentamicin and ciprofloxacin, leading to greater biofilm destruction and bacterial sensitization [278, 279].

Despite advances in preclinical research, the clinical application of phenolic compounds still faces several obstacles. Among the main challenges are the standardization of natural sources, as the phytochemical composition can vary widely depending on the plant species, cultivation conditions, plant part used, and extraction method [280]. Additionally, interindividual variability in response to phenolic, affected by genetic factors, age, sex, gut microbiota, and the patient's health status [281], and the scarcity of robust clinical trials that confirm their efficacy and safety in humans limit their therapeutic recognition. However, innovative strategies have been proposed to overcome these limitations. These include the combination of phenolic compounds with antibiotics to achieve synergistic effects that enhance antimicrobial activity and reduce the necessary doses of conventional drugs; strategic chemical modification, such as derivatization or the use of prodrugs to improve the stability, permeability, and selectivity of phenolics [272, 282], and encapsulation in nanostructured systems such as liposomes, polymeric nanoparticles, and cyclodextrin complexes which provide greater protection for the compounds, increase their bioavailability, and improve targeting to the site of action [283]. These approaches represent promising pathways to enable the safe and effective clinical use of phenolic compounds in combating microbial infections, particularly in light of the growing challenge of antimicrobial resistance.

### 10.1 Synergistic Interactions With Antibiotics

Antibiotics are of immense value in fighting bacterial infections; however, their effectiveness has been increasingly threatened by the rapid emergence of resistant bacteria, becoming a major cause of failure in the treatment of infectious diseases. Recently, there has been a growing number of pathogenic strains displaying multidrug resistance (MDR) phenotypes, such as methicillin‐resistant *S. aureus* (MRSA) and resistant clinical isolates of *E. coli* [284].

In February 2017, in light of increasing antibiotic resistance, the World Health Organization published a list of pathogens designated by the acronym ESKAPE: *Enterococcus faecium, S. aureus, Klebsiella pneumoniae, Acinetobacter baumannii, P*, and *Enterobacter* species. These bacteria are highly virulent and have demonstrated resistance to multiple antibiotics, representing a serious threat to public health [285]. In this scenario, there is an urgent need to seek alternative approaches for the treatment of infections caused by these pathogens. Combating these infections requires the development of new therapeutic strategies that do not rely exclusively on traditional antibiotics. This includes the search for new molecules with novel mechanisms of action, especially those derived from natural products with antimicrobial properties [253, 286].

One promising strategy is the use of synergistic combinations of bioactive compounds with antibiotics, which can enhance or even restore the antimicrobial activity of drugs that have become ineffective due to bacterial resistance [284]. These combinations may improve the interaction of the antibiotic with its target site within the bacterial cell, while the natural compound exerts additional or complementary mechanisms of action [246, 287]. The synergistic approach offers multiple advantages: expansion of the antimicrobial spectrum, prevention of resistance development, and reduced toxicity, as lower concentrations of each agent can be used [284, 288].

Among natural molecules, phenolic compounds have shown considerable potential. These plant‐derived secondary metabolites possess antimicrobial activity against both Gram‐positive and Gram‐negative pathogens and have demonstrated synergistic interactions with antibiotics [286]. Polyphenols can interfere with QS, biofilm formation, and virulence regulation. These mechanisms do not necessarily induce immediate cell death, but can sensitize bacteria to the action of antibiotics [253, 287]. For example, previous studies suggested that biofilm eradication is more effective when combining antibiotics and QS inhibitors compared to using antibiotics alone [246, 271].

An emerging strategy to combat resistant bacteria is the co‐administration of antibiotics with such compounds. Synergistic activity of two compounds is achieved if they have different modes of action and such action results in a greater effect on the bacteria. In this context, some phenolic compounds can disrupt bacterial communication, impair biofilm architecture, or alter motility patterns, thus weakening bacterial defenses and increasing their susceptibility to antibiotics. Additionally, this approach may reduce the dose of antibiotics used [246, 284, 288]. Several in vitro studies have widely used the checkerboard method to assess synergistic effects, which allows for testing multiple concentrations of phenolic compounds in combination with antibiotics in 96‐well plates. Synergy is usually confirmed when the fractional inhibitory concentration index (FICi) is ≤ 0.5 [288].

Numerous studies have reported synergistic effects between phenolics or plant extracts and different classes of antibiotics against sensitive and multidrug‐resistant pathogenic strains. Lima et al. [235] evaluated the synergistic effect of rosmarinic acid, baicalein, curcumin, and resveratrol in combination with tobramycin. The tested combinations proved to be a promising strategy for reducing the required antibiotic dose while maintaining efficacy against *P. aeruginosa* biofilms, as observed in the optical microscope. By checkerboard assay, Vipin et al. [287] observed synergistic interactions between quercetin and the antibiotics tobramycin and amikacin, resulting in significant inhibition of *P. aeruginosa* biofilm formation.

Hossain et al. [288] reported synergism between gallic acid and ampicillin, as well as between hamamelitannin and erythromycin. The authors observed enhanced inhibition of growth, viability, biofilm formation, and motility of *E. coli* when compared to antibiotics alone. Moreover, in the study of Sanhueza et al. [284], the synergy between grape pomace extracts (rich in phenolic compounds) and antibiotics of various classes against MDR clinical isolates of *S. aureus* and *E. coli* was evaluated. The combinations showed strong synergistic effects (FICi values between 0.031 and 0.5). The most abundant phenolic compounds identified in the extract were quercetin (26.3%), gallic acid (24.4%), protocatechuic acid (16.7%), and luteolin (11.4%). Interestingly, no direct correlation was found between relative abundance and synergistic effect, suggesting a multi‐target mode of action. Cytotoxicity testing in HeLa cells confirmed that the combinations were nontoxic at concentrations in which synergism was observed.

Hemaiswarya and Doble [289] studied the interaction of seven phenylpropanoids (cinnamic, p‐coumaric, caffeic, chlorogenic, ferulic, 3,4‐dimethoxycinnamic, and 2,4,5‐trimethoxycinnamic acids) with five antibiotics (amikacin, ampicillin, ciprofloxacin, erythromycin, and vancomycin) against Gram‐negative (*E. coli*, *Enterobacter aerogenes*, *P. aeruginosa*) and Gram‐positive (*S. aureus*) bacteria. Time‐kill assays revealed enhanced bactericidal effects for combinations such as ferulic acid with amikacin. The phenylpropanoids were found to disrupt bacterial membranes, and structure‐activity relationship analysis suggested that hydrophilic substituents enhanced this activity.

Cho et al. [286] demonstrated that kaempferol and quercetin exhibited synergistic effects with ciprofloxacin and rifampicin against *S. aureus* and MRSA. Epigallocatechin gallate, found in Korean green tea, was also synergistic with various β‐lactam antibiotics against MRSA clinical isolates. The combination of tea polyphenols with oxacillin was effective in all MRSA strains tested.

The antibacterial and synergistic effects of a polyphenol‐rich complex of green propolis and olive leaf extracts against *S. aureus*, *Haemophilus influenzae*, and *Klebsiella pneumoniae* were investigated by Ramata‐Stunda et al. [290]. The extracts showed synergy with azithromycin and clarithromycin against *S. aureus*, and other antibiotics against *H. influenzae*. Time‐kill assays confirmed that synergistic effects occurred within the first 6 h of treatment. These results support the use of phenolic‐rich formulations in managing respiratory infections.

In summary, the growing problem of bacterial resistance has made it increasingly difficult to treat many infectious diseases. The findings presented here suggest that phenolic compounds, due to their diverse mechanisms and broad antimicrobial action, are promising candidates to be used alongside conventional antibiotics to improve therapeutic outcomes, reduce side effects, and slow down resistance development.

### 10.2 Combined Application of Phenolic Compounds With Other Antimicrobials (Bacteriocins)

The beneficial properties of different natural antimicrobials are topics of recent studies for improving safety and extending the shelf life of food commodities. Besides the antimicrobial activity of phenolic compounds widely disseminated in literature, bacteriocins, antimicrobial peptides produced by various microorganisms, are also promising candidates for inhibiting undesirable microorganisms. Bacteriocins are a family of antimicrobial peptides produced by various bacterial cultures that can inhibit the growth of other, generally closely related bacteria [291, 292]. However, with recent advances in knowledge about bacteriocins, they are now recognized as more complex antimicrobials, capable of inhibiting and/or killing not only bacteria but also microbial representatives beyond the bacterial domain, including fungi, viruses, and even *Mycobacterium* spp. [293].

Like phenolic compounds, bacteriocins have been suggested as complementary or potential alternatives to antibiotics. Some bacteriocins may positively interact and enhance the antimicrobial activity of antibiotics by increasing membrane permeability, inhibiting efflux pumps, or interfering with resistance mechanisms. Synergistic interactions between bacteriocins and antibiotics have been reported, for example, between pediocin PA‐1 and ciprofloxacin [294], enterocins and ciprofloxacin [295], and mundticin CRL35 with membrane‐acting antibiotics [296].

In recent years, food safety issues caused by foodborne pathogens and spoilage bacteria have become a major public health concern worldwide. Bacteriocins have been widely used as natural preservatives in food systems. Notably, nisin and pediocin PA‐1 are commercially produced and commonly applied in the biopreservation of dairy products [291, 297].

Nisin, produced by *Lactococcus lactis* and first described in 1933, is commercially available and approved as a food additive by both EFSA and the FDA [291]. Pediocin PA‐1, produced by *Pediococcus acidilactici* and also by some strains of *Lactobacillus* [298] and *Lactococcus* [299], is another well‐known bacteriocin. Enterocin AS‐48, produced by *Enterococcus faecalis*, was originally reported by Martínez‐Bueno et al. [300]. These three bacteriocins have been extensively studied and applied in various food matrices, including dairy, meat, and fruit‐based products.

However, when used individually, bacteriocins present limitations such as high costs for isolation and purification, a narrow inhibitory spectrum, enzymatic degradation, and reduced activity in complex food environments [301]. To overcome these challenges, recent studies have demonstrated that co‐treatment with bacteriocins and other antimicrobial substances, including phenolic compounds, essential oils (EOs), plant extracts, and organic acids, can result in synergistic effects against spoilage microorganisms and foodborne pathogens, enhancing food safety [301].

The combination of phenolic compounds and bacteriocins can enhance antimicrobial efficacy, extend the shelf life of food products, and improve control of foodborne pathogens and spoilage bacteria [137]. Several studies have reported successful applications of such combinations in cheese, meat, and fruit products. Bacteriocins and EOs (rich in phenolic compounds, flavonoids, and terpenoids) can inhibit microbial growth by altering membrane permeability or causing membrane disruption, while preserving food quality and sensory acceptance [301, 302]. For instance, Wang et al. [303]. found that nisin combined with *Perilla frutescens* EO disrupted the cell membranes of *S. aureus*, *E. coli*, *Salmonella* Enteritidis, and *Pseudomonas tolaasii*, forming cavities that led to cytoplasmic leakage, cell lysis, and death.

In another study, the fractional inhibitory concentration index (FICi) confirmed a synergistic effect between nisin and carvacrol against *L. monocytogenes* [304]. With the same compound, Li et al. [302] showed the synergistic effect of nisin and carvacrol against *S. aureus* in pasteurized milk, at 25°C and 4°C.

Alves et al. [305] evaluated the synergistic effects of nisin combined with phenolic compounds (carvacrol, thymol, eugenol, and cinnamaldehyde) against *S. aureus* and *L. monocytogenes* in cow milk. All combinations exhibited bacteriostatic activity, with a significant reduction in *L. monocytogenes* counts compared to controls. These findings highlight the potential of such combinations for dairy products preservation.

In meat products, the combined use of bacteriocins and phenolic compounds offers effective protection against spoilage and contamination. As protein‐rich foods, meat products are susceptible to microbial degradation and oxidative processes, leading to undesirable changes in color, flavor, texture, and nutritional value. The incorporation of phenolic compounds with bacteriocins into meat products can inhibit microbial growth and oxidative reactions, thereby extending shelf life [306, 307].

The antibacterial potential of a semi‐purified preparation containing bacteriocins from *Bacillus velezensis*, combined with a mixture of lemongrass and chili spur pepper extracts, was evaluated for controlling the growth of *E. coli* and *S*. Typhimurium in dried seasoned squid over 28 days of storage. The bactericidal activity of the combined bacteriocins and herbal extracts against these foodborne pathogens involved cell lysis, evidenced by ruptured cell walls. This approach offers tremendous advantages as a novel, safe, natural, and effective strategy to enhance the microbial safety of dried seafood products [308].

Fruits and vegetables, which are high in carbohydrates and prone to enzymatic browning and microbial spoilage, can also benefit from such combined treatments. Postharvest applications involving phenolic compounds and organic acids, bacteriocins, or extracts from LAB have shown effectiveness in preserving freshness, reducing browning, and preventing fungal contamination in fruit coatings and treatments [137, 309, 310, 311].

Edible films and coatings made from proteins, polysaccharides, and lipids are promising biodegradable and sustainable alternatives to prolong the shelf life of fresh produce. These materials can be enhanced with bioactive compounds, such as phenolics, bacteriocins, antioxidants, probiotic bacteria, and plant extracts, further improving their protective effects [309, 310]. For instance, Hernández‐Carrilo et al. [311]. evaluated pectin‐based coatings enriched with reuterin (a metabolite produced by *Limosilactobacillus reuteri*) and lemon essential oil on strawberries inoculated with *Penicillium* spp. during 31 days of storage. Coated samples showed a more intense, redder color than control samples, while reuterin decreased viable *Penicillium* spp. more than two logarithmic cycles in comparison to the other treatments. The developed coatings show great potential to prevent spoilage without compromising quality.

The application of LAB and their metabolites in food biopreservation has attracted increasing interest, especially due to their potential to enhance safety and extend shelf life using naturally derived compounds. Among LAB, *Lmb. reuteri* stands out for its heterofermentative metabolism, which enables the production of a variety of antimicrobial substances, including lactic acid, acetic acid, ethanol, bacteriocin (reutericin 6), and reutericyclin, an antimicrobial compound related to tetramic acids [253, 312, 313].

The reuterin system is particularly promising due to its broad‐spectrum antimicrobial activity and notable stability across different food processing conditions [312]. It can be introduced into food matrices either through the direct addition of *Lmb. reuteri* for in situ production, or via incorporation of purified reuterin. However, the latter approach may be subject to regulatory constraints, depending on local legislation. A recent review by Lima et al. [314] highlights the versatile applications of *Lmb. reuteri* in dairy systems, with emphasis on their functional and technological attributes, health‐promoting potential, and efficacy in controlling foodborne pathogens, primarily attributed to reuterin production.

In summary, bacteriocins are generally recognized as safe antimicrobial peptides, traditionally used in food fermentation and preservation, and increasingly explored for therapeutic applications. Similarly, phenolic compounds are widely applied in food processing and traditional medicine due to their antioxidant, anti‐inflammatory, and antimicrobial activities. Despite their promising potential (individually and in combination), the safety and toxicity profiles of both remain underexplored and may vary depending on the compound, dose, and route of administration. Therefore, further studies are needed to ensure their safe and effective use in food and medical applications [137].

### 10.3 Hurdle Technology

The food manufacturing sector has adopted a range of practices and technologies to improve food safety. In recent years, the demand for fresh, minimally processed, and “clean label” foods has intensified, driven by growing consumer awareness of food safety and health considerations. One promising response to these demands has been the implementation of hurdle technology in food processing [315].

Hurdle technology is a preservation strategy based on the combination of multiple sublethal treatments or antimicrobial agents to inhibit microbial growth while maintaining product quality and nutritional value. Instead of relying on a single intense intervention, hurdle technology uses synergistic or additive effects among different “hurdles”, such as low pH, reduced water activity, refrigeration, and the application of natural antimicrobials like bacteriocins and phenolic compounds, to create an inhospitable environment for pathogens and spoilage microorganisms [315, 316].

This multi‐target approach is particularly promising for the development of clean‐label food products, as it enables a reduction in the use of synthetic additives or severe processing conditions. In this context, the integration of natural antimicrobials with physical treatments (e.g., mild heat or high‐pressure processing) can further enhance microbial control while preserving sensory quality [316, 317]. The use of natural compounds, including microbial metabolites, plant‐derived extracts, and animal‐origin antimicrobials, has demonstrated broad‐spectrum activity against foodborne pathogens and spoilage organisms, improving both food safety and shelf life.

Biopreservatives produced by beneficial bacteria, such as lactic acid, bacteriocins, and reuterin, can delay lipid oxidation, prevent color loss, extend storage time, and ensure food safety. Importantly, these compounds should exhibit antimicrobial activity without negatively affecting the consumer's gastrointestinal microbiota. Some phenolic compounds can interact with membrane proteins, altering their structure and function. This is related to disruptions in electron transport, nutrient uptake, nucleic acid and protein synthesis, and enzymatic activity [317, 318].

In summary, hurdle technology offers a strategic framework for combining natural antimicrobials and mild preservation methods to ensure food safety, extend shelf life, and meet consumer expectations for high‐quality products. Its success will depend on identifying optimal hurdle combinations that are effective against diverse pathogens while maintaining the sensory and nutritional properties of foods.

## Conflicts of Interest

The authors declare no conflicts of interest.

## Data Availability

Data sharing not applicable to this article as no datasets were generated or analysed during the current study.
